# *q*-Rung orthopair fuzzy 2-tuple linguistic WASPAS algorithm for patients’ prioritization based on prioritized Maclaurin symmetric mean aggregation operators

**DOI:** 10.1038/s41598-024-57452-w

**Published:** 2024-05-09

**Authors:** Fatima Abbas, Jawad Ali, Wali Khan Mashwani, Necla Gündüz, Muhammad I. Syam

**Affiliations:** 1 Institute of Numerical Sciences, Kohat University of Science and Technology, Kohat, KPK Pakistan; 2 Department of Statistics, Faculty of Science, Gazi University, Ankara, Turkey; 3 Department of Mathematical Sciences, United Arab Emirates University, P. O. Box 15551, Al-Ain, UAE

**Keywords:** *q*-Rung orthopair fuzzy 2-tuple linguistic term set, Maclaurin symmetric mean, Prioritized averaging, WASPAS, MCGDM, Engineering, Mathematics and computing

## Abstract

Due to the fuzziness of the medical field, *q*-rung orthopair fuzzy 2-tuple linguistic (*q*-RF2L) set is the privileged way to aid medical professionals in conveying their assessments in the patient prioritization problem. The theme of the present study is to put forward a novel approach centered around the merging of prioritized averaging (PA) and the Maclaurin symmetric mean (MSM) operator within *q*-RF2L context. According to the prioritization of the professionals and the correlation among the defined criteria, we apply both PA and MSM to assess priority degrees and relationships, respectively. Keeping the pluses of the PA and MSM operators in mind, we introduce two aggregation operators (AOs), namely *q*-RF2L prioritized Maclaurin symmetric mean and *q*-RF2L prioritized dual Maclaurin symmetric mean operators. Meanwhile, some essential features and remarks of the proposed AOs are discussed at length. Based on the formulated AOs, we extend the weighted aggregated sum product assessment methodology to cope with *q*-RF2L decision-making problems. Ultimately, to illustrate the practicality and effectiveness of the stated methodology, a real-world example of patients’ prioritization problem is addressed, and an in-depth analysis with prevailing methods is performed.

## Introduction

The global population is increasing, but healthcare resources are limited, posing challenges for healthcare institutions in meeting public health needs. Prioritizing timely patient treatment is crucial, yet these institutions grapple with efficiently allocating scarce medical resources among patients^1,2^. Ghanaian public healthcare facilities face a similar dilemma, as the country’s population growth has led to a rise in emergency cases, while the availability of sick beds remains insufficient. The shortage of sick beds in public healthcare institutions has sparked debates among various healthcare stakeholders in Ghana. Research by Sun et al.^3^ shows that limited medical resources often result in extended waiting times and treatment delays, increasing the likelihood of patients developing complications and, in some cases, leading to fatalities^4^. Numerous scholars^2,3,5^ emphasize the crucial importance of prioritizing patients promptly for effective casualty treatment. This involves addressing several complex research questions in the context of multi-criteria group decision-making (MCGDM): How can we prioritize patients in situations where decision-making involves uncertainty and imprecision? What strategies can be employed to address patient prioritization when multiple experts with priority relationships are involved? How do we tackle patient prioritization when there are interdependencies among multiple input factors and priority relationships among evaluation criteria?

Numerous prior studies^2–4,6,7^ have approached the issue of patient prioritization from diverse angles. For instance, Ashour and Kremer^6^ introduced a dynamic patient grouping and prioritization method to enhance emergency department efficiency. Zhang et al.^7^ employed fuzzy multi-criteria decision-making (MCDM) techniques to investigate patient prioritization, utilizing a hesitant fuzzy linguistic-VIekriterijumsko KOmpromisno Rangiranje (VIKOR) method. Similarly, Zhang et al.^2^ proposed an intuitionistic multiplicative rangement et synthèse de données relarionnelles (ORESTE) method for hospitalization patient prioritization. Davodabadi et al.^4^ developed a fuzzy-technique for order of preference by similarity to ideal solution (TOPSIS) method with discrete event simulation for ICU patient prioritization. Nonetheless, these studies did not delve into the crucial aspect of evaluating the interdependence of criteria in real-world decision-making (DM). In contrast, Sun et al.^3^ addressed patient prioritization using the hesitant fuzzy linguistic projection-based multi-attributive border approximation area comparison (MABAC) method and enhanced the traditional MABAC method by incorporating the Bonferroni mean to account for the relationship between two input factors. Nevertheless, these models do not tackle the interrelationships among multiple input factors. In sum, existing research has developed models for patient prioritization, but none have comprehensively addressed all the research challenges outlined above. Hence, there is a pressing need to develop an appropriate technique to effectively handle patient prioritization for hospital admissions in healthcare institutions while considering interdependencies.

DM processes frequently encounter inherent uncertainty and vagueness^8–11^. Based on their level of expertise, health experts may hesitate in their assessments and opt to employ fuzzy data sets to express patient evaluations for hospital admission. To assist experts in making informed decisions, Attansov^12^ introduced the concept of intuitionistic fuzzy sets (IFS). IFS enables experts to convey their expressions by incorporating both membership grade (MG) and non-membership grade (NMG), providing a more nuanced representation of their assessments. Szmidt and Kacprzyk^13^ pioneered the exploration of medical applications using IFSs. Subsequently, Yager^14^ developed the Pythagorean fuzzy set (PyFS) concept. PyFS models exhibit greater robustness than IFS models in handling practical applications^15–18^. However, both models encounter limitations. For instance, when DM scenarios involve opinions like (0.8, 0.9), both PyFSs and IFSs demonstrate an inability to apply their methodologies effectively. To address these limitations, Yager introduced^19^ a more advanced and generalized form of fuzzy set, termed the *q*-rung orthopair fuzzy set (*q*-ROFS). This variant stands out because it incorporates the parameter ‘*q*,’ which significantly expands the information space. Consequently, various methods have been formulated and refined within the *q*-ROFS framework by researchers^20–23^. Akram and Shahzadi^20^ advanced the notion of q-ROFS by developing a series aggregation operators (AOs) grounded in Yager’s norm. They also thoroughly explored and provided numerical evidence to support the relevant characteristics of these operators. Seikh and Mandal^21^ established an MCDM method utilizing their Frank-operations-based AOs in a q-rung orthopair fuzzy (q-ROF) environment with unknown weights. Recently, Kumar and Gupta^24^ addressed the challenge of project selection with their innovative DM algorithm, distinguished by its ability to incorporate confidence levels for the alternatives. Numerous other scholars have also introduced novel AOs and their implementations in the context of MCDM^25–28^.

The 2-tuple linguistic (2TL) learning approach, known for its efficacy in minimizing information distortion and loss, was first conceptualized by scholars in^29^. This paradigm, integral for dealing with human language in DM, has led to the development of numerous methodologies and 2TL AOs. In this realm, Deng et al.^30^ introduced generalized 2TL Pythagorean fuzzy Heronian mean AOs, integrating generalized Heronian mean with 2TL Pythagorean fuzzy numbers. Wei and Gao^31^ further contributed by proposing Pythagorean fuzzy 2TL power AOs, merging power geometric and average operations with Pythagorean fuzzy 2TL data, aimed at resolving MCDM issues. Wei et al.^32^ expanded this framework by introducing the q-rung orthopair fuzzy 2TL (q-RF2L) sets, allowing the expression of MG and NMG of elements in relation to 2TL variables, along with operational laws. They also developed various q-RF2L Heronian mean AOs. Abbas et al.^33^ pioneered a clustering algorithm that processes data in the q-RF2L format, adeptly handling situations with unspecified weight information. Complementing the advancements of q-RF2L set, Ju et al.^34^ formulated a strategy for tackling MCGDM problems using q-RF2L input. This strategy includes q-RF2L weighted averaging and geometric operators and the novel q-RF2L Muirhead mean and dual Muirhead mean operators. However, all these existing AOs within the q-RF2L framework fall short in addressing scenarios where criteria and experts simultaneously exhibit interdependence and varying priorities. This gap is particularly relevant in real-world situations, such as prioritizing patient admissions in hospitals.

Maclaurin^35^ initially introduced the Maclaurin symmetric mean (MSM), later expanded by Detemple and Robertson^36^. MSM effectively models the interconnections among multiple inputs provided by experts. This concept led to Qin and Liu^37^ proposing the dual Maclaurin symmetric mean (DMSM), which, alongside MSM, has garnered significant attention in the field of information aggregation. For instance, Mu et al.^38^ introduced an interval-valued Pythagorean fuzzy power MSM operator for MCGDM, and Ali^39^ developed partitioned MSM operators using hesitant fuzzy numbers. However, many MSM and DMSM operators overlook the prioritization of experts and criteria. Addressing this, Yager^40^ proposed the prioritized averaging (PA) operator. Ali^41^ then utilized PA’s prioritizing capabilities to create probabilistic hesitant bipolar fuzzy Hamacher prioritized AOs for DM. Similarly, Akram et al.^42^ explored MCGDM with spherical fuzzy prioritized weighted AOs, and Rong et al.^43^ applied complex q-RF2L MSM and DMSM operators to emergency program selection. Despite these advancements, the PA operator has not yet been adapted to the q-RF2L context. Given the strengths of PA, MSM, and DMSM, there’s a clear opportunity to integrate these operators, developing new q-RF2L AOs for MCGDM methods that can effectively tackle prioritized DM challenges.

From the discussions and analyses previously outlined, the motivations for this study can be summarized as follows:

- The *q*-RF2L theory represents a hybrid model for information representation, skillfully integrating the notable aspects of *q*-ROFS and the 2-tuple linguistic model into a cohesive framework. This model allows for a precise representation of assessment values within predefined LTS in uncertain environments. Despite the distinct attributes of the q-RF2LS, to date, only a few scholarly works^32–34^ explore this context.
- Information fusion is crucial in consolidating the preference information of decision experts. Moreover, many practical issues require consideration of the correlation between identified criteria while also necessitating the modeling of priority relationships. However, the current *q*-RF2L AOs lack the capability to effectively manage such scenarios, rendering them inadequate for numerous practical applications.
- The WASPAS method is renowned for its ability to assess and rank alternatives with a heightened level of reliability, as evidenced in various studies^44–46^. Given these strengths, there’s a compelling need to expand the WASPAS approach into the q-RF2L setting. This extension would not only leverage the method’s inherent strengths but also enhance its applicability in more complex and uncertain environments to capitalize further on its assessment and ranking capabilities in this advanced context.
- Taking into account the benefits of *q*-RF2LS, it is likely that health experts would favor this framework for patient assessment. The WASPAS method has demonstrated its efficacy in DM for such issues. However, there is a noticeable research gap in applying this method within a *q*-RF2L environment specifically for addressing the problem of patient prioritization.

Motivated by the considerations mentioned above, this paper aims to devise an innovative decision-making approach for evaluating patient prioritization issues. To fulfill this goal, we initially develop appropriate AOs to aggregate q-RF2L information. Subsequently, our focus shifts to constructing a decision algorithm that can effectively prioritize patients in emergency departments. Finally, it is essential to validate the effectiveness and advantages of the proposed decision algorithm from various perspectives. Consequently, the specific aims of this article are outlined as follows:

- To introduce two innovative AOs, namely q-RF2LPMSM and q-RF2LPDMSM, designed to account for the interdependence among criteria while simultaneously modeling their priority relationships.
- To thoroughly elucidate the characteristics of the proposed operators, focusing on its monotonicity and boundedness, and to detail special cases supported by rigorous proof.
- To devise an MCGDM method that integrates the WASPAS technique with the proposed operators. This method aims to address the traditional limitation of WASPAS by considering the interdependence of evaluation criteria in its decision process while also factoring in the priority relationships among these criteria.
- To showcase the practicality of the proposed framework by applying it to solve the problem of prioritizing patients for hospital admission.

## Preliminaries

This section mainly addresses several basic ideas and operators regarding LTS, *q*-RFLS, MSM, and DMSM operators.

Suppose  represents an LTS with an odd cardinality. Any label,  signifies the possible value for a linguistic variable, and it has to adhere with the stipulations^29,47^ listed below:

1. *Ordered set*:
2. *Negation operator*: , such that
3. *Max operator*:  if
4. *Min operator*: Min operator:  if

For instance, *S* can be defined as

$$\begin{aligned} S=\left\{ \begin{array}{c} {\rrbracket }_0 = \text {extremely poor}, {\rrbracket }_1 = \text {very poor}, {\rrbracket }_2 = \text {poor}, {\rrbracket }_3 = \text {medium}, {\rrbracket }_4 = \text {good}, {\rrbracket }_5 = \text {very good},\\ {\rrbracket }_6 = \text {extremely good} \end{array}\right\} . \end{aligned}$$

In light of the concept of symbolic translation, Herrera and Martinez^29,47^ set up the 2-tuple fuzzy linguistic representation approach. It is employed to convey linguistic assessment information as a 2-tuple  where  is a linguistic label from the pre-defined linguistic term set *S*, $\vartriangle _{}$ is the measure of symbolic translation, and $\vartriangle _{}\in [-0.5, 0.5)$.

### Definition 1^29,47^

 Let $\vartheta$ be the calculated result of an aggregation of the indices of a set of labels assessed in an LTS *S*, i.e., the outcome of a symbolic aggregation operation, $\vartheta \in \left[ 0,\ell \right]$, with $\ell$ being the cardinality of *S*. If $r = round(\vartheta )$ and $\vartriangle _{}=\vartheta -r$ are two numbers such that $r\in \left[ 0,\ell \right]$ and $\vartriangle _{} \in [-0.5, 0.5)$, then $\vartriangle _{}$ is known as a symbolic translation.

### Definition 2^29,47^

 Let  be an LTS and $\vartheta \in \left[ 0,\ell \right]$ be a numerical value representing the linguistic symbolic aggregation outcome. Then, the function $\curlywedge$ that retrieves the 2-tuple linguistic information equivalent to $\vartheta$ is then described as

1 $$\begin{aligned}{} & {} \curlywedge : \left[ 0, \ell \right] \longrightarrow S \times [-0.5, 0.5){{,}} \end{aligned}$$

2 $$\begin{aligned}{} & {} \curlywedge \left( \vartheta \right) ={\left\{ \begin{array}{ll} {\rrbracket }_r, &{} r=round\left( \vartheta \right) \\ \vartriangle _{}=\vartheta -r, &{} \vartriangle _{} \in [-0.5, 0.5). \end{array}\right. } \end{aligned}$$

where round $\left( .\right)$ is the conventional round function, ${\rrbracket }_r$ is the index label closest to $\vartheta$, and $\vartriangle _{}$ is the symbolic translation value.

### Definition 3^29,47^

 Let  be an LTS and $\left( {\rrbracket }_r, \vartriangle \right)$ be a 2-tuple. There is always a function $\curlyvee$ can be described, such that, from a 2-tuple $\left( {\rrbracket }_r, \vartriangle \right)$ it yield its equivalent numerical value $\vartheta \in \left[ 1, \ell \right]$, which is

3 $$\begin{aligned}{} & {} \curlyvee : S \times [-0.5, 0.5) \longrightarrow \left[ 0, \ell \right] {{,}} \end{aligned}$$

4 $$\begin{aligned}{} & {} \curlyvee \left( {\rrbracket }_r,\vartriangle \right) =r+\vartriangle =\vartheta . \end{aligned}$$

### Definition 4^32^

 A *q*-RFLS $\mathcal {F}$ on a universal set $\texttt {Z}$ is described as

5 $$\begin{aligned} \mathcal {F}=\left\{ \left( \left( {\rrbracket }_{r(z_i)},\vartriangle (z_i)\right) ,\left\langle {\mathfrak {m}}(z_i), {\mathfrak {n}}(z_i)\right\rangle \right) \right\} , \end{aligned}$$

where ${\rrbracket }_{r(z_i)} \in S$, $\vartriangle \left( z_i\right) \in [-0.5,0.5)$, ${\mathfrak {m}}(z_i),{\mathfrak {n}}(z_i) \in [0,1]$, with the restriction $0\le {\mathfrak {m}}^{q}(z_i)+{\mathfrak {n}}^{q}(z_i)\le 1$ $\left( {q}\ge 1 \right)$ $\forall \; z_i\in \texttt {Z}$. The numbers ${\mathfrak {m}}(z_i),{\mathfrak {n}}(z_i)$ symbolize the MG and NMG of the element $z_i$ to linguistic variable $\left( {\rrbracket }_{r(z_i)},\vartriangle (z_i)\right)$, respectively. Furthermore, $\pi _{\mathcal {F}}\left( z_i \right) =1-\left( {\mathfrak {m}}^{q}(z_i)+{\mathfrak {n}}^{q}(z_i) \right) ^{1/{q}}$ is named refusal grade, and the *q*-rung orthopair fuzzy 2-tuple linguistic number (*q*-RFLN) is marked by $\partial =\left( \left( {\rrbracket }_{r},\vartriangle \right) ,\left\langle {\mathfrak {m}}, {\mathfrak {n}}\right\rangle \right) .$

### Definition 5^32^

 Let $\partial _1=\left( \left( {\rrbracket }_{{r}_1},{\vartriangle }_1\right) ,\left\langle {{\mathfrak {m}}}_1, {{\mathfrak {n}}}_1\right\rangle \right)$ and $\partial _2=\left( \left( {\rrbracket }_{{r}_2},{\vartriangle }_2\right) ,\left\langle {{\mathfrak {m}}}_2, {{\mathfrak {n}}}_2\right\rangle \right)$ be two *q*-RFLNs. Then, their basic operational rules are listed as:

1. $$\partial _1\oplus \partial _2=\left( \curlywedge \left( \curlyvee \left( {\rrbracket }_{{r}_1},{\vartriangle }_1\right) +\curlyvee \left( {\rrbracket }_{{r}_2},{\vartriangle }_2\right) \right) , \left\langle \left( {{\mathfrak {m}}}^{q}_1+{{\mathfrak {m}}}^{q}_2-{{\mathfrak {m}}}^{q}_1{{\mathfrak {m}}}^{q}_2\right) ^{1/{q}},{{\mathfrak {n}}}_1{{\mathfrak {n}}}_2\right\rangle \right) ;$$
2. $$\partial _1\otimes \partial _2=\left( \curlywedge \left( \curlyvee \left( {\rrbracket }_{{r}_1},{\vartriangle }_1\right) \cdot \curlyvee \left( {\rrbracket }_{{r}_2},{\vartriangle }_2\right) \right) , \left\langle {{\mathfrak {m}}}_1{{\mathfrak {m}}}_2,\left( {{\mathfrak {n}}}^{q}_1+{{\mathfrak {n}}}^{q}_2-{{\mathfrak {n}}}^{q}_1{{\mathfrak {n}}}^{q}_2\right) ^{1/{q}}\right\rangle \right) ;$$
3. $$\alpha \partial _1=\left( \curlywedge \left( \alpha \curlyvee \left( {\rrbracket }_{{r}_1},{\vartriangle }_1\right) \right) , \left\langle \left( 1-\left( 1-{{\mathfrak {m}}}^{q}_1\right) ^\alpha \right) ^{1/{q}},{{\mathfrak {n}}}^{\alpha }_1 \right\rangle \right) ~~ \alpha > 0;$$
4. $$\partial ^{\alpha }_1=\left( \curlywedge \left( \left( \curlyvee \left( {\rrbracket }_{{r}_1},{\vartriangle }_1\right) \right) ^{\alpha }\right) ,\left\langle {{\mathfrak {m}}}^{\alpha }_1,\left( 1-\left( 1-{{\mathfrak {n}}}^{q}_1\right) ^\alpha \right) ^{1/{q}}\right\rangle \right) ~~ \alpha > 0;$$
5. $$\partial ^c_1=\left( \left( {\rrbracket }_{{r}_1},{\vartriangle }_1\right) ,\left\langle {{\mathfrak {n}}}_1, {{\mathfrak {m}}}_1\right\rangle \right) .$$

### Definition 6^32^

 Let $\partial _1=\left( \left( {\rrbracket }_{{r}_1},{\vartriangle }_1\right) ,\left\langle {{\mathfrak {m}}}_1, {{\mathfrak {n}}}_1\right\rangle \right)$ be a *q*-RFLN. Then its score function ${\text{\t{S}}\left(\partial_1\right)}$ and accuracy function ${\text{\t{A}}\left(\partial_1\right)}$ are described as:

6 $$\begin{aligned}{} & {} {\text{\t{S}}\left(\partial_1\right)}=\frac{\curlyvee \left( {\rrbracket }_{{r}_1},{\vartriangle }_1\right) \cdot \left( 1+{{\mathfrak {m}}}^{q}_1-{{\mathfrak {n}}}^{q}_1 \right) }{2\ell }, \end{aligned}$$

7 $$\begin{aligned}{} & {} {\text{\t{A}}\left(\partial_1\right)}=\curlyvee \left( {\rrbracket }_{{r}_1},{\vartriangle }_1\right) \cdot \left( {{\mathfrak {m}}}^{q}_1+{{\mathfrak {n}}}^{q}_1 \right) . \end{aligned}$$

### Definition 7^32^

 Let $\partial _1=\left( \left( {\rrbracket }_{{r}_1},{\vartriangle }_1\right) ,\left\langle {{\mathfrak {m}}}_1, {{\mathfrak {n}}}_1\right\rangle \right)$ and $\partial _2=\left( \left( {\rrbracket }_{{r}_2},{\vartriangle }_2\right) ,\left\langle {{\mathfrak {m}}}_2, {{\mathfrak {n}}}_2\right\rangle \right)$ be two *q*-RFLNs. Then, they can be compared according to the following laws:

1. If $${\text{\t{S}}\left(\partial_1\right)}>{\text{\t{S}}\left(\partial_2\right)},$$ then $$\partial _1\succ \partial _2$$;
2. If $${\text{\t{S}}\left(\partial_1\right)}={\text{\t{S}}\left(\partial_2\right)},$$ then:
  (i) if $${\text{\t{A}}\left(\partial_1\right)}>{\text{\t{A}}\left(\partial_2\right)},$$ then $$\partial _1\prec \partial _2$$;
  (ii) if ${\text{\t{A}}\left(\partial_1\right)}={\text{\t{A}}\left(\partial_2\right)},$ then $\partial _1= \partial _2.$

### Definition 8^48^

 Let $\partial _1=\left( \left( {\rrbracket }_{{r}_1},{\vartriangle }_1\right) ,\left\langle {{\mathfrak {m}}}_1, {{\mathfrak {n}}}_1\right\rangle \right)$ and $\partial _2=\left( \left( {\rrbracket }_{{r}_2},{\vartriangle }_2\right) ,\left\langle {{\mathfrak {m}}}_2, {{\mathfrak {n}}}_2\right\rangle \right)$ be two *q*-RF2LNs. Then the Hamming distance between $\partial _1$ and $\partial _2$ is defined as

8 $$\begin{aligned} d\left( \partial _1,\partial _2 \right) =\frac{1}{2 \ell }\left[ \left| \left( 1+{{\mathfrak {m}}}^{q}_1-{{\mathfrak {n}}}^{q}_1 \right) .\curlyvee \left( {\rrbracket }_{r_1},{\vartriangle }_1 \right) - \left( 1+{{\mathfrak {m}}}^{q}_2-{{\mathfrak {n}}}^{q}_2 \right) .\curlyvee \left( {\rrbracket }_{r_2},{\vartriangle }_2 \right) \right| \right] . \end{aligned}$$

### Definition 9^40^

 Let €={€$_1$,€$_2....$€$_n$} be a collection of criteria with a prioritization relationship €$_1 \succ$€$_2 \succ ....\succ$ €$_n$, where $\succ$ means “higher priority”. The value €$_j(a)$ represents the criterion value of the alternative *a* with respect to criteria €$_j$ and satisfies the condition €$_j(a)\in [0,1]$. If9then PA is called the prioritized averaging operator, where ,  and $T_1=1$.

### Definition 10^35^

 Let ${\mathscr {N}}_i$ $(i=1,2, \ldots ,{{\scalebox{0.7}{\text{\saturn}}}})$ be a set of non-negative numbers, then the MSM operator is characterized as follows:10where  is a parameter and  are  integer values taken from the collection $\left\{ 1,2,\ldots ,{{\scalebox{0.7}{\text{\saturn}}}} \right\}$ of ${{\scalebox{0.7}{\text{\saturn}}}}$ integer values,  represent the binomial coefficient.

The MSM operator exhibits the following characteristics:

if $${\mathscr {N}}_i={\mathscr {N}}\left( i=1,2, \ldots {{\scalebox{0.7}{\text{\saturn}}}}\right) ;$$
if ${\mathscr {N}}_i\le \check{{\mathscr {N}}}_i$ for all *i*.

According to the MSM operator’s definition, Qin and Liu^37^ proposed its dual form, as articulated below.

### Definition 11^37^

 Let ${\mathscr {N}}_i$ $(i=1,2, \ldots ,{{\scalebox{0.7}{\text{\saturn}}}})$ be a set of non-negative numbers, then the DMSM operator is characterized as follows:11where  is a parameter and  are  integer values taken from the collection $\left\{ 1,2, \ldots ,{{\scalebox{0.7}{\text{\saturn}}}} \right\}$ of ${{\scalebox{0.7}{\text{\saturn}}}}$ integer values,  represent the binomial coefficient.

Similarly to the MSM operator, the DMSM operator also exhibits the aforementioned characteristics.

## *q*-RF2L prioritized aggregation operators

This section introduces the concept of the *q*-RF2LPMSM operator and the *q*-RF2LPDMSM operator. We delve into a comprehensive discussion of other appealing properties inherent to these operators, such as boundary and monotonicity, along with a detailed exploration of their specific instances.

### *q*-RF2L prioritized Maclaurin symmetric mean aggregation operators

Considering the context provided by Definitions 9 and 10, the definition of the *q*-RF2LPMSM operator is presented as follows:

#### Definition 12

Let $\partial _{i}= \left( \left( {\rrbracket }_{{r}_i},{\varkappa }_i\right) ,\left\langle {{\mathfrak {m}}}_i, {{\mathfrak {n}}}_i\right\rangle \right) \left( i=1,2, \ldots ,{{\scalebox{0.7}{\text{\saturn}}}} \right)$ be a family of *q*-RF2LNs, then *q*-rung orthopair fuzzy 2-tuple linguistic prioritized Maclaurin symmetric mean (*q*-RF2LPMSM) is given by12where  , $(i=2, \ldots ,{{\scalebox{0.7}{\text{\saturn}}}})$, $\mathcal {T}_1=1$, ${\text{\t{S}}\left(\partial_t\right)}$ is the score function value of $\partial _t$ and  are  integer values taken from the collection $\left\{ 1,2, \ldots ,{{\scalebox{0.7}{\text{\saturn}}}} \right\}$ of ${{\scalebox{0.7}{\text{\saturn}}}}$ integer values,  represent the binomial coefficient.

#### Theorem 1

Let $\partial _{i}= \left( \left( {\rrbracket }_{{r}_i},{\varkappa }_i\right) ,\left\langle {{\mathfrak {m}}}_i, {{\mathfrak {n}}}_i\right\rangle \right) \left( i=1,2, \ldots ,{{\scalebox{0.7}{\text{\saturn}}}} \right)$ be a family of *q*-RF2LNs, where , then the aggregated value by using the *q*-RF2LPMSM operator is also a *q*-RF2LN, and13where  and , $(i=2, \ldots ,{{\scalebox{0.7}{\text{\saturn}}}})$, $\mathcal {T}_1=1$, ${\text{\t{S}}\left(\partial_t\right)}$ is the score function value of $\partial _t$ and  are  integer values taken from the collection $\left\{ 1,2, \ldots ,{{\scalebox{0.7}{\text{\saturn}}}} \right\}$ of ${{\scalebox{0.7}{\text{\saturn}}}}$ integer values,  represent the binomial coefficient.

#### Proof

By using operational laws, we formulate the aggregated results of *q*-RF2LPMSM as follows: 
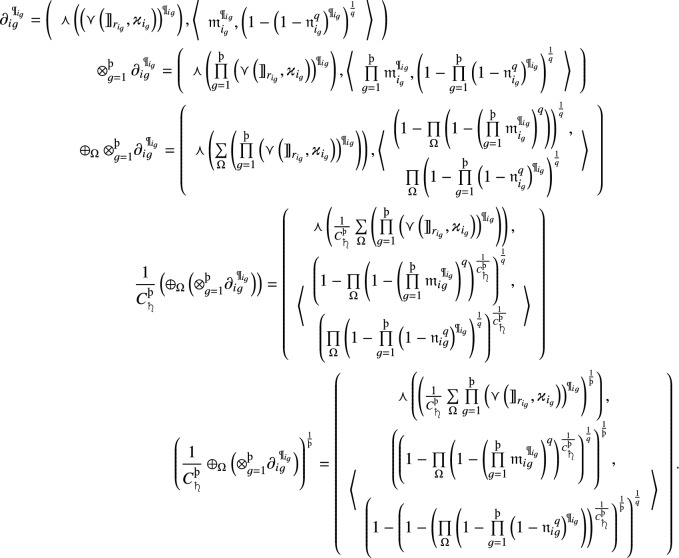


$$\square $$

#### Theorem 2

Let $\partial _{i}= \left( \left( {\rrbracket }_{{r}_i},{\varkappa }_i\right) ,\left\langle {{\mathfrak {m}}}_i, {{\mathfrak {n}}}_i\right\rangle \right) \left( i=1,2, \ldots ,{{\scalebox{0.7}{\text{\saturn}}}} \right)$ and $\breve{{\partial }_{i}}= \left( \left( \breve{{\rrbracket }}_{{r}_{i}},\breve{{{\varkappa }}_i}\right) ,\left\langle \breve{{{{\mathfrak {m}}}}_i}, \breve{{{{\mathfrak {n}}}}_i}\right\rangle \right)$ $\left( i=1,2, \ldots ,{{\scalebox{0.7}{\text{\saturn}}}} \right)$ be two collections of *q*-RF2LNs, if $\partial _i\le {\breve{\partial _i}}$,$\forall i$. Then

14 $$\begin{aligned} {q}-RF2LPMSM(\partial _1,\partial _2,...,\partial _{{\scalebox{0.7}{\text{\saturn}}}})\le {q}-RF2LPMSM(\breve{\partial _1},\breve{\partial _2},...,\breve{\partial _{{\scalebox{0.7}{\text{\saturn}}}}}). \end{aligned}$$

#### Proof

Because $\partial _i\le {\breve{\partial }_i}$, then we have $\left( {\rrbracket }_{{r}_i},{{\varkappa }_i}\right) \le \left( \breve{{\rrbracket }_{{r}_i}},{\breve{{\varkappa }}_i}\right) ,{{\mathfrak {m}}}_i\le \breve{{{\mathfrak {m}}}_i}$ and ${{\mathfrak {n}}}_i\ge \breve{{{\mathfrak {n}}}}_i$ $\forall (i=1,2, \ldots ,{{\scalebox{0.7}{\text{\saturn}}}})$. Then 

Further, since ${{\mathfrak {m}}}_i\le {\breve{{\mathfrak {m}}}}_i$, we have
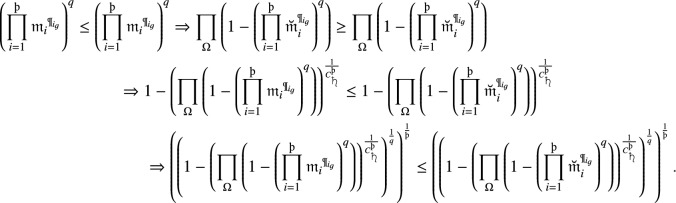


Similar to the testify of non-membership degrees, we attain 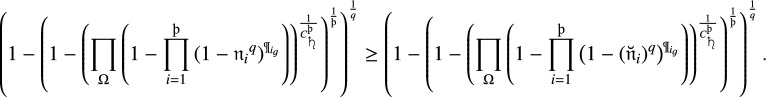


From the above inequalities, we attain

$$\begin{aligned} {q}-RF2LPMSM(\partial _1,\partial _2,...,\partial _{{\scalebox{0.7}{\text{\saturn}}}})\le {q}-RF2LPMSM(\breve{\partial }_1,\breve{\partial }_2,...,\breve{\partial }_{{\scalebox{0.7}{\text{\saturn}}}}). \end{aligned}$$

That proves the monotonicity of q-RF2LPMSM operator. $\square$

#### Theorem 3

Let $\partial _i (i=1,2, \ldots ,{{\scalebox{0.7}{\text{\saturn}}}})$ be a family of *q*-RF2LNs. If $\partial _{i}^{+}=\left( \max \limits _{1\le i\le {{\scalebox{0.7}{\text{\saturn}}}}}\left( {\rrbracket }_{{r}_i},{\vartriangle }_i\right) ,\left\langle \max \limits _{1\le i\le {{\scalebox{0.7}{\text{\saturn}}}}} {{\mathfrak {m}}}_i, \min \limits _{1\le i\le {{\scalebox{0.7}{\text{\saturn}}}}} {{\mathfrak {n}}}_i\right\rangle \right)$ and $\partial _{i}^{-}=\left( \min \limits _{1\le i\le {{\scalebox{0.7}{\text{\saturn}}}}}\left( {\rrbracket }_{{r}_i},{\vartriangle }_i\right) ,\left\langle \min \limits _{1\le i\le {{\scalebox{0.7}{\text{\saturn}}}}} {{\mathfrak {m}}}_i, \max \limits _{1\le i\le {{\scalebox{0.7}{\text{\saturn}}}}} {{\mathfrak {n}}}_i\right\rangle \right)$, then

15 $$\begin{aligned} {q}-RF2LPMSM(\partial ^{-}_1,\partial ^{-}_2,...,\partial ^{-}_{{\scalebox{0.7}{\text{\saturn}}}}) \le {q}-RF2LPMSM(\partial _1,\partial _2,...,\partial _{{\scalebox{0.7}{\text{\saturn}}}}) \le {q}-RF2LPMSM(\partial ^{+}_1,\partial ^{+}_2,...,\partial ^{+}_{{\scalebox{0.7}{\text{\saturn}}}}). \end{aligned}$$

#### Proof

According to Theorem 1, we have 
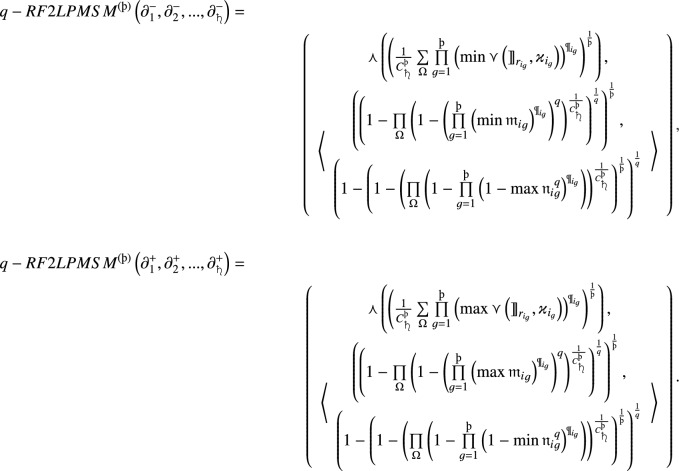


From Theorem 2, we have

$${q}-RF2LPMSM(\partial ^{-}_1,\partial ^{-}_2,...,\partial ^{-}_{{\scalebox{0.7}{\text{\saturn}}}}) \le {q}-RF2LPMSM(\partial _1,\partial _2,...,\partial _{{\scalebox{0.7}{\text{\saturn}}}}) \le {q}-RF2LPMSM(\partial ^{+}_1,\partial ^{+}_2,...,\partial ^{+}_{{\scalebox{0.7}{\text{\saturn}}}}).$$

$$\square $$

In the next, several novel operators shall be attained through assigning diverse values of .

1. If , the *q*-RF2LPMSM operator is yielded to *q*-RF2LP averaging (*q*-RF2LPA) operator, displayed as below: 16
2. If , the *q*-RF2LPMSM operator is yielded to *q*-RF2LP Bonferroni mean (*q*-RF2LPBM) operator, displayed as below: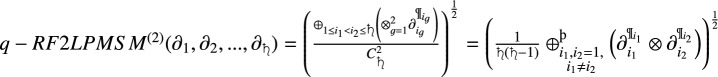  17
3. If , the *q*-RF2LPMSM operator is yielded to *q*-RF2LP generalized Bonferroni mean (*q*-RF2LPGBM) operator, displayed as below: 18
4. If , the *q*-RF2LPMSM operator is yielded to *q*-RF2LP geometric mean (*q*-RF2LPGM) operator, displayed as below: 19

### $\textsf {{q}}$-RF2L prioritized dual Maclaurin symmetric mean aggregation operators

#### Definition 13

Let $\partial _{i}= \left( \left( {\rrbracket }_{{r}_i},{\varkappa }_i\right) ,\left\langle {{\mathfrak {m}}}_i, {{\mathfrak {n}}}_i\right\rangle \right) \left( i=1,2, \ldots ,{{\scalebox{0.7}{\text{\saturn}}}} \right)$ be a family of *q*-RF2LNs, then *q*-rung orthopair fuzzy 2-tuple linguistic prioritized dual Maclaurin symmetric mean ($\textsf {{q}}$-RF2LPDMSM) is given by20where , $(i=2, \ldots ,{{\scalebox{0.7}{\text{\saturn}}}})$, $\mathcal {T}_1=1$, ${\text{\t{S}}\left(\partial_t\right)}$ is the score function value of $\partial _t$ and  are  integer values taken from the collection $\left\{ 1,2, \ldots ,{{\scalebox{0.7}{\text{\saturn}}}} \right\}$ of ${{\scalebox{0.7}{\text{\saturn}}}}$ integer values,  represent the binomial coefficient.

#### Theorem 4

Let $\partial _{i}= \left( \left( {\rrbracket }_{{r}_i},{\varkappa }_i\right) ,\left\langle {{\mathfrak {m}}}_i, {{\mathfrak {n}}}_i\right\rangle \right) \left( i=1,2, \ldots ,{{\scalebox{0.7}{\text{\saturn}}}} \right)$ be a family of *q*-RF2LNs, and , then the aggregated value by using the *q*-RF2LPDMSM operator is also a *q*-RF2LN, and21where  and , $(i=2, \ldots ,{{\scalebox{0.7}{\text{\saturn}}}})$, $\mathcal {T}_1=1$, ${\text{\t{S}}\left(\partial_t\right)}$ is the score function value of $\partial _t$ and  are  integer values taken from the collection $\left\{ 1,2, \ldots ,{{\scalebox{0.7}{\text{\saturn}}}} \right\}$ of ${{\scalebox{0.7}{\text{\saturn}}}}$ integer values,  represent the binomial coefficient.

#### Proof

By using operational laws, we formulate the aggregated results of *q*-RF2LPDMSM as follows: 
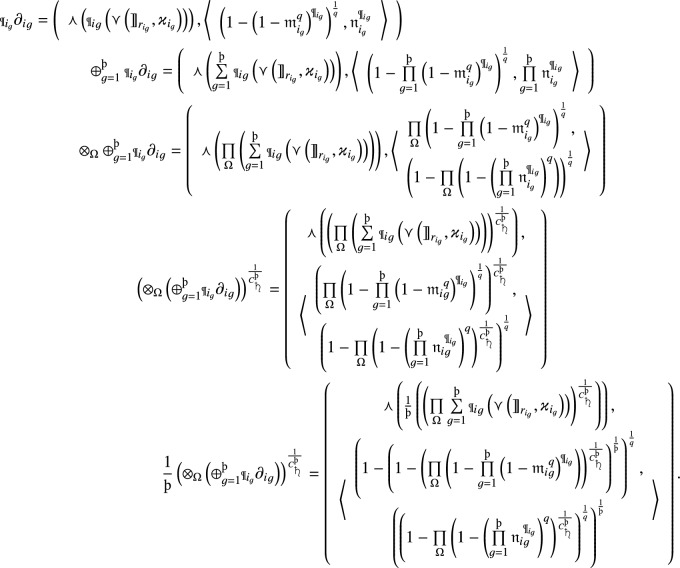


$$\square $$

#### Theorem 5

Let $\partial _{i}= \left( \left( {\rrbracket }_{{r}_i},{\varkappa }_i\right) ,\left\langle {{\mathfrak {m}}}_i, {{\mathfrak {n}}}_i\right\rangle \right) \left( i=1,2, \ldots ,{{\scalebox{0.7}{\text{\saturn}}}} \right)$ and $\breve{{\partial }_{i}}= \left( \left( \breve{{\rrbracket }}_{{r}_{i}},\breve{{{\varkappa }}_i}\right) ,\left\langle \breve{{{{\mathfrak {m}}}}_i}, \breve{{{{\mathfrak {n}}}}_i}\right\rangle \right)$ $\left( i=1,2, \ldots ,{{\scalebox{0.7}{\text{\saturn}}}} \right)$ be two collections of *q*-RF2LNs, if $\partial _i\le {\breve{\partial _i}}$,$\forall i$. Then

22 $$\begin{aligned} {q}-RF2LPDMSM(\partial _1,\partial _2,...,\partial _{{\scalebox{0.7}{\text{\saturn}}}})\le {q}-RF2LPDMSM(\breve{\partial _1},\breve{\partial _2},...,\breve{\partial _{{\scalebox{0.7}{\text{\saturn}}}}}). \end{aligned}$$

#### Proof

Because $\partial _i\le {\breve{\partial }_i}$, then we have $\left( {\rrbracket }_{{r}_i},{{\varkappa }_i}\right) \le \left( \breve{{\rrbracket }_{{r}_i}},{\breve{{\varkappa }}_i}\right) ,{{\mathfrak {m}}}_i\le \breve{{{\mathfrak {m}}}_i}$ and ${{\mathfrak {n}}}_i\ge \breve{{{\mathfrak {n}}}}_i$ $\forall (i=1,2, \ldots ,{{\scalebox{0.7}{\text{\saturn}}}})$. Then 

Further, since ${{\mathfrak {m}}}_i\le {\breve{{\mathfrak {m}}}}_i$, we have
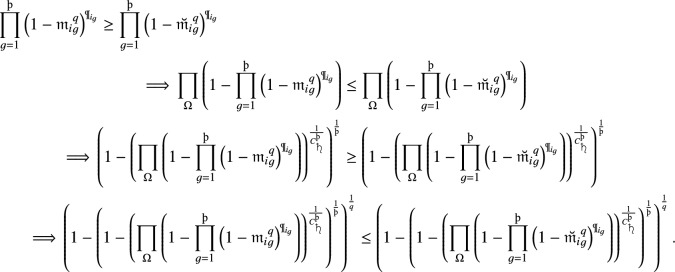


Similarly, for non-membership grades, we have 

From the above inequalities, we get

$$\begin{aligned} {q}-RF2LPDMSM(\partial _1,\partial _2,...,\partial _{{\scalebox{0.7}{\text{\saturn}}}})\le {q}-RF2LPDMSM(\breve{\partial }_1,\breve{\partial }_2,...,\breve{\partial }_{{\scalebox{0.7}{\text{\saturn}}}}). \end{aligned}$$

That proves the monotonicity of q-RF2LPDMSM operator. $\square$

#### Theorem 6

Let $\partial _i (i=1,2, \ldots ,{{\scalebox{0.7}{\text{\saturn}}}})$ be a family of *q*-RF2LNs. If $\partial _{i}^{+}=\left( \max \limits _{1\le i\le {{\scalebox{0.7}{\text{\saturn}}}}}\left( {\rrbracket }_{{r}_i},{\vartriangle }_i\right) ,\left\langle \max \limits _{1\le i\le {{\scalebox{0.7}{\text{\saturn}}}}} {{\mathfrak {m}}}_i, \min \limits _{1\le i\le {{\scalebox{0.7}{\text{\saturn}}}}} {{\mathfrak {n}}}_i\right\rangle \right)$ and $\partial _{i}^{-}=\left( \min \limits _{1\le i\le {{\scalebox{0.7}{\text{\saturn}}}}}\left( {\rrbracket }_{{r}_i},{\vartriangle }_i\right) ,\left\langle \min \limits _{1\le i\le {{\scalebox{0.7}{\text{\saturn}}}}} {{\mathfrak {m}}}_i, \max \limits _{1\le i\le {{\scalebox{0.7}{\text{\saturn}}}}} {{\mathfrak {n}}}_i\right\rangle \right)$, then

23 $$\begin{aligned} {q}-RF2LPDMSM(\partial ^{-}_1,\partial ^{-}_2,...,\partial ^{-}_{{\scalebox{0.7}{\text{\saturn}}}}) \le {q}-RF2LPDMSM(\partial _1,\partial _2,...,\partial _{{\scalebox{0.7}{\text{\saturn}}}}) \le {q}-RF2LPDMSM(\partial ^{+}_1,\partial ^{+}_2,...,\partial ^{+}_{{\scalebox{0.7}{\text{\saturn}}}}). \end{aligned}$$

#### Proof

According to Theorem 4, we have 
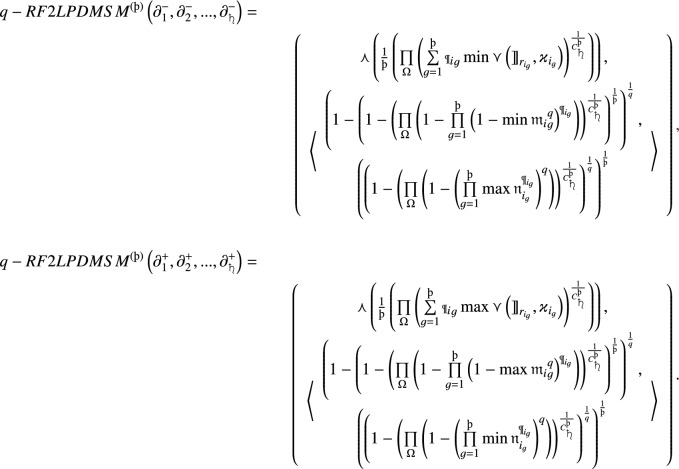


From Theorem 5, we have

$${q}-RF2LPDMSM(\partial ^{-}_1,\partial ^{-}_2,...,\partial ^{-}_{{\scalebox{0.7}{\text{\saturn}}}}) \le {q}-RF2LPMSM(\partial _1,\partial _2,...,\partial _{{\scalebox{0.7}{\text{\saturn}}}}) \le {q}-RF2LPDMSM(\partial ^{+}_1,\partial ^{+}_2,...,\partial ^{+}_{{\scalebox{0.7}{\text{\saturn}}}}).$$ $$\square $$

A few novel operators shall be attained through assigning diverse parameter values of .

1. If , the *q*-RF2LPDMSM operator is yielded to *q*-RF2L prioritized geometric mean (${q}-RF2LPGM$) operator, displayed as below: 24
2. If , the *q*-RF2LPDMSM operator is yielded to *q*-RF2L prioritized geometric Bonferroni mean (*q*-RF2LPGBM) operator, displayed as below: 25
3. If , the *q*-RF2LPDMSM operator is yielded to *q*-RF2L prioritized generalized geometric Bonferroni mean (*q*-RF2LPGGBM) operator, displayed as below: 26
4. If , the *q*-RF2LPDMSM operator is yielded to *q*-RF2L prioritized averaging mean (*q*-RF2LAM) operator, displayed as below: 27

## A novel MCGDM approach with $\textsf {{q}}$-RF2L information

This section elaborates on a novel approach called the *q*-RF2LP MCGDM method. We extend the existing WASPAS technique to accommodate situations that involve *q*-rung orthopair fuzzy 2-tuple linguistic contexts. Furthermore, we improve the WASPAS method by integrating the *q*-RF2LPMSM and *q*-RF2LPDMSM operators. These enhancements are made to efficiently tackle *q*-RF2LP MCGDM challenges.

### Description of the MCGDM problem

Let’s consider a scenario where we have a finite set of alternatives, denoted as ${{\text{\Shilling}}} = \left\{ {{\text{\Shilling}}}_1, {{\text{\Shilling}}}_2, ..., {{\text{\Shilling}}}_m\right\}$, and a set of criteria, denoted as €= {€$_1$, €$_2,...,$ €$_{{\scalebox{0.7}{\text{\saturn}}}}\}$. These criteria come with priority information indicating their relative importance, where €$_1>$ €$_2 > ...>$ €$_{{\scalebox{0.7}{\text{\saturn}}}}$, signifying that criterion  holds a higher priority than criterion €$_\zeta$ if . Additionally, we have a group of decision-makers (DMs), denoted as ${{\text{\OE}}} = {{{\text{\OE}}}_1, {{\text{\OE}}}_2, ..., {{\text{\OE}}}_p}$, and they are ranked in a linear order of importance: ${{\text{\OE}}}_1> {{\text{\OE}}}_2> ...> {{\text{\OE}}}_p$. In this ranking, DM ${{\text{\OE}}}_z$ holds a higher priority than DM ${{\text{\OE}}}_t$ if $z < t$. Each DM is tasked with expressing their viewpoints using *q*-RF2LNs: , which represents the *q*-RF2L evaluations of the alternatives concerning the criteria, provided by the expert ${{\text{\OE}}}^{(t)}$. Thus, the *q*-RF2LP matrix can be constructed as follows: where  symbolizes *q*-RFOLN provided by *t*th DM to which alternative ${{\text{\Shilling}}}_i$ meets that the criteria  having the constraint that .

### WASPAS approach with $\textsf {{q}}$-RF2LNs

Considering that the individual decision matrix $M^{(t)}$ includes both benefit and cost criteria, it is essential to normalize these criteria. This normalization process involves converting the cost-type criteria into benefit-type criteria, which can be accomplished using the following equation:28where  represents the complement of .

Using Definition 9 and the normalized individual decision matrix  as a basis, we proceed to compute the priority degrees among the experts in the following manner:29As per the setup of the MCGDM problem, it is necessary to aggregate the individual normalized decision matrices into a collective decision matrix. This collective decision matrix can be generated using either the *q*-RF2LPMSM or *q*-RF2LPDMSM operator. To calculate the aggregated value of alternative “${{\text{\Shilling}}}_i$” concerning the criteria “”, we use the Eq. (13) or Eq. (21) in the following manner:30where 31Based on the outcomes from *q*-RF2LPMSM and *q*-RF2LPDMSM operators, we derive an integrated group decision matrix denoted as . The construction of the decision matrix *M* is accomplished as: 

The element  in the decision matrix *M* represents the collective assessment of alternative ${{\text{\Shilling}}}_i$ concerning criterion . Regarding Definition 6 and the collective decision matrix *M*, the process of determining the priority levels among the criteria is performed in the following manner:32Using the foundational concept of the WASPAS method and the collective decision matrix *M*, we compute the measures of the *q*-RF2L prioritized sum model (*q*-RF2L-PSM) $\bowtie ^{(1)}_i$ for each alternative ${{\text{\Shilling}}}_i$. This calculation involves applying the *q*-RF2LPMSM operator in the following manner:33Similarly, we ascertain the measures of the *q*-RF2L prioritized product model (*q*-RF2L-PPM) $\bowtie ^{(2)}_i$ for each alternative ${{\text{\Shilling}}}_i$ using the *q*-RF2LPDMSM operator in the following manner:34Hence, according to Definition 6, the scores ${\text{\t{S}}(\bowtie^{(1)}_i)}$ and ${\text{\t{S}}(\bowtie^{(2)}_i)}$ in Eqs. (33) and (34) can be determined, respectively. Additionally, we obtain the combined measure of the *q*-RF2LPWASPAS method $\bowtie _i$ for each alternative by merging the measures of *q*-RF2L-PSM and *q*-RF2L-PPM. This result is calculated using the following formula:35where $\theta$ represents the aggregating coefficient of decision precision, and $\theta \in [0, 1]$. Finally, we rank the alternatives in descending order based on the value of $\bowtie _i$.

### Decision making procedure

This section is devoted to outlining an approach based on utilizing the *q*-RF2LPMSM, *q*-RF2LPDMSM, and the WASPAS method. The key steps of this novel decision-making process are presented as follows:

*Step 1*: Considering the real-world decision-making scenario, we establish the set of feasible alternatives denoted by ${{\text{\Shilling}}} = \{{{\text{\Shilling}}}_1, {{\text{\Shilling}}}_2,..., {{\text{\Shilling}}}_m\}$ and the set of evaluation criteria represented by € = {€$_1$, €$_2,...,$ €$_{{\scalebox{0.7}{\text{\saturn}}}}\}$, along with their respective priority information. Let ${{\text{\OE}}} =\{{{\text{\OE}}}_1, {{\text{\OE}}}_2, ..., {{\text{\OE}}}_p\}$ be the group of DMs, with a specific prioritization among them. Consequently, we generate the *q*-rung orthopair fuzzy 2-tuple linguistic decision matrix , provided by expert ${{\text{\OE}}}^{(t)}$.
*Step 2*: By the application of Eq. (28), we standardize the individual decision matrix $M^{(t)}$ by ($i = 1, 2, \ldots , m$; ).
*Step 3*: Utilizing Eq. (29), we determine the priority degrees among the DMs.
*Step 4*: Once the value of  is given, we employ *q*-RF2LPMSM and Eq. (30) or *q*-RF2LPDMSM and Eq. (31) to aggregate the normalized individual decision matrices into a unified collective decision matrix. Then, we construct the collective decision matrix .
*Step 5*: Based on Eq. (32), we calculate the priority degrees among the criteria.
*Step 6*: Using Eq. (33), we compute the measures of *q*-RF2LPSM $\bowtie ^{(1)}_i$ for each alternative ${{\text{\Shilling}}}_i$.
*Step 7*: Using Eq. (34), we calculate the measures of *q*-RF2LPPM $\bowtie ^{(2)}_i$ for each alternative ${{\text{\Shilling}}}_i$.
*Step 8*: Next, we apply Eq. (35) to obtain the aggregated measure of WASPAS $\bowtie _i$ for each alternative ${{\text{\Shilling}}}_i$.
*Step 9*: Finally, we rank all the alternatives in descending order based on the $\bowtie _i$ values.

**Figure 1:**
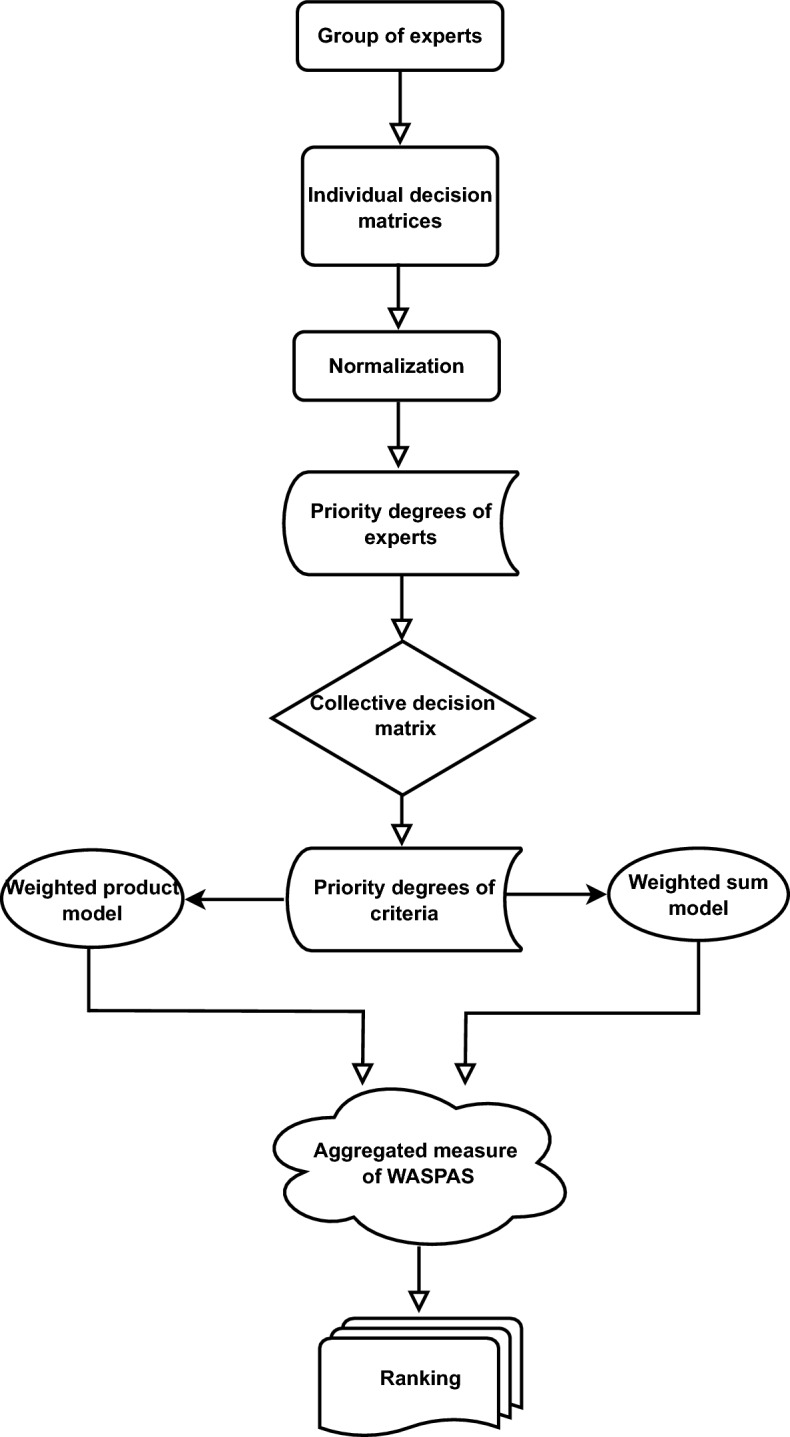
Graphical illustration of the developed MCGDM approach.

The developed MCGDM approach is visually illustrated in Fig. 1.

## Application

In this section, we implement the proposed method to address the prioritization problem of patients in a prominent teaching hospital in Ghana. Furthermore, we perform sensitivity and comparison analyses to showcase the validity and reliability of our proposed approach.

### Background

Ghana’s growing population has led to an increase in health-related cases in recent years, posing significant challenges for healthcare institutions like the Komfo Anokye Teaching Hospital (KATH), especially with the limited availability of beds. To enhance the patient admission process, KATH established an inpatient Admission Service Unit (ASU) aimed at optimizing hospital admissions and ensuring efficient allocation of medical resources. This initiative is particularly focused on promptly admitting patients with severe illnesses. Previously, the hospital relied on an artificial screening process for patient admission, which was flawed due to human intervention in bed allocation and the complexity of decision-making, as noted in^2^. The process involved balancing conflicting factors and managing uncertainty in information. To improve the efficiency of the ASU, we are implementing the *q*-rung orthopair fuzzy 2-tuple linguistic WASPAS method to prioritize patient admissions effectively. Three health experts from the ASU, denoted as ${{\text{\OE}}}= \left\{ {{\text{\OE}}}_1, {{\text{\OE}}}_2, {{\text{\OE}}}_3\right\}$, will assess patients post-initial examination by doctors, with a priority order of ${{\text{\OE}}}_1>{{\text{\OE}}}_2>{{\text{\OE}}}_3$. The evaluation criteria adopted by the experts, based on studies from^2,3,7^, and relevant to Ghana’s context, include disease severity, emergency degree, priority for special diseases, and type of medical insurance. These criteria are prioritized as €$_1>$ €$_2>$ €$_3>$ €$_4$. To manage the inherent uncertainty of information, the health experts will use *q*-RF2LTNs to provide their preference values for various alternatives ${{\text{\Shilling}}}_i (i = 1, 2, 3, 4, 5)$ relative to the criteria . The resulting *q*-rung orthopair fuzzy 2-tuple linguistic decision matrices from these evaluations are presented in Tables 1, 2, and 3.

### Method implementation process

*Step 1* The initial evaluation matrices provided by three DMs ${{\text{\OE}}}_1, {{\text{\OE}}}_2$ and ${{\text{\OE}}}_3$ are listed in Tables 1,2, and 3, respectively.

**Table 1:** *q*-RF2L decision matrix $M^{(1)}$.

	€$_1$	€$_2$	€$_3$	€$_4$
${{\text{\Shilling}}}_1$	$\left( \left( {\rrbracket }_{2},0\right) ,\left\langle 0.6, 0.3\right\rangle \right)$	$\left( \left( {\rrbracket }_{3},0\right) ,\left\langle 0.4, 0.5\right\rangle \right)$	$\left( \left( {\rrbracket }_{5},0\right) ,\left\langle 0.5, 0.4\right\rangle \right)$	$\left( \left( {\rrbracket }_{4},0\right) ,\left\langle 0.6, 0.3\right\rangle \right)$
${{\text{\Shilling}}}_2$	$\left( \left( {\rrbracket }_{1},0\right) ,\left\langle 0.5, 0.4\right\rangle \right)$	$\left( \left( {\rrbracket }_{3},0\right) ,\left\langle 0.6, 0.4\right\rangle \right)$	$\left( \left( {\rrbracket }_{2},0\right) ,\left\langle 0.7, 0.3\right\rangle \right)$	$\left( \left( {\rrbracket }_{4},0\right) ,\left\langle 0.8, 0.7\right\rangle \right)$
${{\text{\Shilling}}}_3$	$\left( \left( {\rrbracket }_{2},0\right) ,\left\langle 0.7, 0.1\right\rangle \right)$	$\left( \left( {\rrbracket }_{1},0\right) ,\left\langle 0.8, 0.3\right\rangle \right)$	$\left( \left( {\rrbracket }_{4},0\right) ,\left\langle 0.4, 0.1\right\rangle \right)$	$\left( \left( {\rrbracket }_{5},0\right) ,\left\langle 0.6, 0.1\right\rangle \right)$
${{\text{\Shilling}}}_4$	$\left( \left( {\rrbracket }_{1},0\right) ,\left\langle 0.3, 0.4\right\rangle \right)$	$\left( \left( {\rrbracket }_{3},0\right) ,\left\langle 0.7, 0.1\right\rangle \right)$	$\left( \left( {\rrbracket }_{4},0\right) ,\left\langle 0.5, 0.5\right\rangle \right)$	$\left( \left( {\rrbracket }_{2},0\right) ,\left\langle 0.5, 0.1\right\rangle \right)$
${{\text{\Shilling}}}_5$	$\left( \left( {\rrbracket }_{4},0\right) ,\left\langle 0.5, 0.4\right\rangle \right)$	$\left( \left( {\rrbracket }_{2},0\right) ,\left\langle 0.4, 0.3\right\rangle \right)$	$\left( \left( {\rrbracket }_{1},0\right) ,\left\langle 0.6, 0.2\right\rangle \right)$	$\left( \left( {\rrbracket }_{3},0\right) ,\left\langle 0.7, 0.3\right\rangle \right)$

**Table 2:** *q*-RF2L decision matrix $M^{(2)}$.

	€$_1$	€$_2$	€$_3$	€$_4$
${{\text{\Shilling}}}_1$	$\left( \left( {\rrbracket }_{5},0\right) ,\left\langle 0.6, 0.5\right\rangle \right)$	$\left( \left( {\rrbracket }_{6},0\right) ,\left\langle 0.4, 0.3\right\rangle \right)$	$\left( \left( {\rrbracket }_{2},0\right) ,\left\langle 0.5, 0.5\right\rangle \right)$	$\left( \left( {\rrbracket }_{3},0\right) ,\left\langle 0.6, 0.3\right\rangle \right)$
${{\text{\Shilling}}}_2$	$\left( \left( {\rrbracket }_{4},0\right) ,\left\langle 0.5, 0.4\right\rangle \right)$	$\left( \left( {\rrbracket }_{3},0\right) ,\left\langle 0.6, 0.3\right\rangle \right)$	$\left( \left( {\rrbracket }_{1},0\right) ,\left\langle 0.7, 0.3\right\rangle \right)$	$\left( \left( {\rrbracket }_{6},0\right) ,\left\langle 0.6, 0.3\right\rangle \right)$
${{\text{\Shilling}}}_3$	$\left( \left( {\rrbracket }_{5},0\right) ,\left\langle 0.6, 0.4\right\rangle \right)$	$\left( \left( {\rrbracket }_{3},0\right) ,\left\langle 0.7, 0.5\right\rangle \right)$	$\left( \left( {\rrbracket }_{6},0\right) ,\left\langle 0.9, 0.1\right\rangle \right)$	$\left( \left( {\rrbracket }_{1},0\right) ,\left\langle 0.8, 0.2\right\rangle \right)$
${{\text{\Shilling}}}_4$	$\left( \left( {\rrbracket }_{2},0\right) ,\left\langle 0.4, 0.3\right\rangle \right)$	$\left( \left( {\rrbracket }_{1},0\right) ,\left\langle 0.6, 0.5\right\rangle \right)$	$\left( \left( {\rrbracket }_{3},0\right) ,\left\langle 0.7, 0.2\right\rangle \right)$	$\left( \left( {\rrbracket }_{5},0\right) ,\left\langle 0.8, 0.1\right\rangle \right)$
${{\text{\Shilling}}}_5$	$\left( \left( {\rrbracket }_{5},0\right) ,\left\langle 0.5, 0.3\right\rangle \right)$	$\left( \left( {\rrbracket }_{2},0\right) ,\left\langle 0.6, 0.3\right\rangle \right)$	$\left( \left( {\rrbracket }_{6},0\right) ,\left\langle 0.8, 0.1\right\rangle \right)$	$\left( \left( {\rrbracket }_{5},0\right) ,\left\langle 0.8, 0.2\right\rangle \right)$

**Table 3:** *q*-RF2L decision matrix $M^{(3)}$.

	€$_1$	€$_2$	€$_3$	€$_4$
${{\text{\Shilling}}}_1$	$\left( \left( {\rrbracket }_{1},0\right) ,\left\langle 0.5, 0.4\right\rangle \right)$	$\left( \left( {\rrbracket }_{3},0\right) ,\left\langle 0.6, 0.5\right\rangle \right)$	$\left( \left( {\rrbracket }_{4},0\right) ,\left\langle 0.7, 0.1\right\rangle \right)$	$\left( \left( {\rrbracket }_{5},0\right) ,\left\langle 0.8, 0.4\right\rangle \right)$
${{\text{\Shilling}}}_2$	$\left( \left( {\rrbracket }_{2},0\right) ,\left\langle 0.6, 0.1\right\rangle \right)$	$\left( \left( {\rrbracket }_{1},0\right) ,\left\langle 0.6, 0.1\right\rangle \right)$	$\left( \left( {\rrbracket }_{3},0\right) ,\left\langle 0.5, 0.4\right\rangle \right)$	$\left( \left( {\rrbracket }_{4},0\right) ,\left\langle 0.9, 0.1\right\rangle \right)$
${{\text{\Shilling}}}_3$	$\left( \left( {\rrbracket }_{4},0\right) ,\left\langle 0.7, 0.4\right\rangle \right)$	$\left( \left( {\rrbracket }_{5},0\right) ,\left\langle 0.8, 0.6\right\rangle \right)$	$\left( \left( {\rrbracket }_{1},0\right) ,\left\langle 0.1, 0.2\right\rangle \right)$	$\left( \left( {\rrbracket }_{6},0\right) ,\left\langle 0.4, 0.1\right\rangle \right)$
${{\text{\Shilling}}}_4$	$\left( \left( {\rrbracket }_{5},0\right) ,\left\langle 0.8, 0.3\right\rangle \right)$	$\left( \left( {\rrbracket }_{1},0\right) ,\left\langle 0.9, 0.1\right\rangle \right)$	$\left( \left( {\rrbracket }_{2},0\right) ,\left\langle 0.6, 0.2\right\rangle \right)$	$\left( \left( {\rrbracket }_{3},0\right) ,\left\langle 0.5, 0.5\right\rangle \right)$
${{\text{\Shilling}}}_5$	$\left( \left( {\rrbracket }_{6},0\right) ,\left\langle 0.8, 0.5\right\rangle \right)$	$\left( \left( {\rrbracket }_{2},0\right) ,\left\langle 0.7, 0.4\right\rangle \right)$	$\left( \left( {\rrbracket }_{1},0\right) ,\left\langle 0.6, 0.5\right\rangle \right)$	$\left( \left( {\rrbracket }_{4},0\right) ,\left\langle 0.6, 0.6\right\rangle \right)$

*Step 2* Referring to Eq. (28), it is required to normalize the individual decision matrices $M^{(1)}$ to $M^{(3)}$. However, since all the criteria are benefit criteria, normalization is unnecessary.

*Step 3* Utilizing the information provided in Tables 1, 2 and 3 and Eq. (29), we compute the priority degrees  among the DMs, and the results are given below: 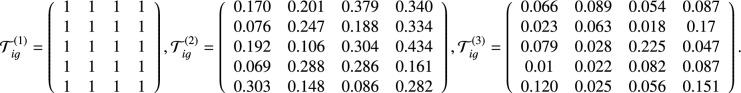


Next, we will use the *q*-RF2LPMSM and *q*-RF2LFPDMSM operators for further analysis. In what follows, we begin by addressing the problem using the *q*-RF2LPMSM operator, followed by applying the *q*-RF2LPDMSM operator.

#### Solving problem via the WASPAS method with PMSM operator

In this part, we utilize the q-RF2L PMSM operator to integrate information from individual decision matrices ranging from $M^{(1)}$ to $M^{(3)}$.

Step 4: Taking , we use Eq. (30) to determine the overall preference value of alternative ${{\text{\Shilling}}}_i$ in relation to  to form the collective decision matrix depicted in Table 4.

**Table 4:** Aggregated decision matrix obtained via q-RF2L PMSM operator.

	€$_1$	€$_2$	€$_3$	€$_4$
${{\text{\Shilling}}}_1$	$\left( \left( {\rrbracket }_{1},0.3168\right) ,\left\langle 0.8796, 0.2415\right\rangle \right)$	$\left( \left( {\rrbracket }_{2},-0.4661\right) ,\left\langle 0.8171,0.2991\right\rangle \right)$	$\left( \left( {\rrbracket }_{2},-0.3446\right) ,\left\langle 0.8326, 0.2924\right\rangle \right)$	$\left( \left( {\rrbracket }_{2},-0.3901\right) ,\left\langle 0.8735, 0.2093\right\rangle \right)$
${{\text{\Shilling}}}_2$	$\left( \left( {\rrbracket }_{1},0.0381\right) ,\left\langle 0.8929, 0.2348\right\rangle \right)$	$\left( \left( {\rrbracket }_{1},0.4579\right) ,\left\langle 0.8749, 0.2295\right\rangle \right)$	$\left( \left( {\rrbracket }_{1},0.2364\right) ,\left\langle 0.9162, 0.1942\right\rangle \right)$	$\left( \left( {\rrbracket }_{2},-0.3328\right) ,\left\langle 0.9185, 0.3314\right\rangle \right)$
${{\text{\Shilling}}}_3$	$\left( \left( {\rrbracket }_{1},0.3495\right) ,\left\langle 0.9011, 0.1420\right\rangle \right)$	$\left( \left( {\rrbracket }_{1},0.0451\right) ,\left\langle 0.9455, 0.2347\right\rangle \right)$	$\left( \left( {\rrbracket }_{2},-0.4277\right) ,\left\langle 0.8303, 0.0867\right\rangle \right)$	$\left( \left( {\rrbracket }_{2},-0.4513\right) ,\left\langle 0.8946, 0.095\right\rangle \right)$
${{\text{\Shilling}}}_4$	$\left( \left( {\rrbracket }_{1},0.0195\right) ,\left\langle 0.8822, 0.2127\right\rangle \right)$	$\left( \left( {\rrbracket }_{1},0.3674\right) ,\left\langle 0.8965, 0.1637\right\rangle \right)$	$\left( \left( {\rrbracket }_{2},-0.4036\right) ,\left\langle 0.8709, 0.2378\right\rangle \right)$	$\left( \left( {\rrbracket }_{1},0.3364\right) ,\left\langle 0.8838, 0.1307\right\rangle \right)$
${{\text{\Shilling}}}_5$	$\left( \left( {\rrbracket }_{2},-0.3250\right) ,\left\langle 0.8453, 0.2623\right\rangle \right)$	$\left( \left( {\rrbracket }_{1},0.2793\right) ,\left\langle 0.9129, 0.1944\right\rangle \right)$	$\left( \left( {\rrbracket }_{1},0.0501\right) ,\left\langle 0.9153, 0.1633\right\rangle \right)$	$\left( \left( {\rrbracket }_{2},-0.4619\right) ,\left\langle 0.9048, 0.246\right\rangle \right)$

*Step 5* Based on Table 4, we calculate the priority degrees  for the set of criteria. The outcomes are computed as follows (Table 5): 

*Step 6* Using Eq. (33), along with , we calculate the prioritized sum model for each alternative ${{\text{\Shilling}}}_i$ ($i = 1, 2, 3, 4, 5$), as shown in Table 6.

*Step 7* Similarly, applying Eq. (34), , we compute the prioritized product model for each alternative, also presented in Table 6.

*Step 8* To obtain the combined measure $\bowtie _i$ for each alternative, we utilize Eq. (35) and display the outcomes in Table 6.

*Step 9* Based on the values of $\bowtie _i$(i = 1, 2, 3, 4, 5), we establish the following ranking for the alternatives: ${{\text{\Shilling}}}_5>{{\text{\Shilling}}}_3> {{\text{\Shilling}}}_1> {{\text{\Shilling}}}_4> {{\text{\Shilling}}}_2$. As a result, the most significant patient is ${{\text{\Shilling}}}_5$.

#### Solving problem via the WASPAS method with PDMSM operator

In the present part, we consider the q-RF2L PDMSM operator to derive the integrated information from individual decision matrices ranging from $M^{(1)}$ to $M^{(3)}$.

*Step 4* Fixing , we apply Eq. (31) to compute the overall preference value of each alternative ${{\text{\Shilling}}}_i$ concerning . These preference values are then consolidated to form the collective decision matrix, illustrated in Table 5.

**Table 5:** Aggregated decision matrix obtained via q-RF2L PDMSM operator.

	€$_1$	€$_2$	€$_3$	€$_4$
${{\text{\Shilling}}}_1$	$\left( \left( {\rrbracket }_{1},-0.2865\right) ,\left\langle 0.3898, 0.8256\right\rangle \right)$	$\left( \left( {\rrbracket }_{1},0.0642\right) ,\left\langle 0.2885,0.8077\right\rangle \right)$	$\left( \left( {\rrbracket }_{1},0.0774\right) ,\left\langle 0.3526, 0.7835\right\rangle \right)$	$\left( \left( {\rrbracket }_{1},0.1164\right) ,\left\langle 0.4331, 0.7414\right\rangle \right)$
${{\text{\Shilling}}}_2$	$\left( \left( {\rrbracket }_{0},0.3563\right) ,\left\langle 0.3106, 0.8493\right\rangle \right)$	$\left( \left( {\rrbracket }_{1},-0.1986\right) ,\left\langle 0.4045, 0.7517\right\rangle \right)$	$\left( \left( {\rrbracket }_{0},0.4180\right) ,\left\langle 0.4625, 0.7943\right\rangle \right)$	$\left( \left( {\rrbracket }_{1},0.3896\right) ,\left\langle 0.5618, 0.7780\right\rangle \right)$
${{\text{\Shilling}}}_3$	$\left( \left( {\rrbracket }_{1},-0.1934\right) ,\left\langle 0.4527, 0.7893\right\rangle \right)$	$\left( \left( {\rrbracket }_{0},0.3735\right) ,\left\langle 0.4948, 0.8701\right\rangle \right)$	$\left( \left( {\rrbracket }_{1},0.2081\right) ,\left\langle 0.4136, 0.5964\right\rangle \right)$	$\left( \left( {\rrbracket }_{1},-0.0810\right) ,\left\langle 0.4799, 0.6492\right\rangle \right)$
${{\text{\Shilling}}}_4$	$\left( \left( {\rrbracket }_{0},0.2796\right) ,\left\langle 0.2220, 0.8658\right\rangle \right)$	$\left( \left( {\rrbracket }_{1},-0.4433\right) ,\left\langle 0.4649, 0.7863\right\rangle \right)$	$\left( \left( {\rrbracket }_{1},0.0039\right) ,\left\langle 0.3963, 0.7482\right\rangle \right)$	$\left( \left( {\rrbracket }_{1},-0.2407\right) ,\left\langle 0.4009, 0.7173\right\rangle \right)$
${{\text{\Shilling}}}_5$	$\left( \left( {\rrbracket }_{1},0.3417\right) ,\left\langle 0.3833, 0.7711\right\rangle \right)$	$\left( \left( {\rrbracket }_{1},-0.4962\right) ,\left\langle 0.3107, 0.8071\right\rangle \right)$	$\left( \left( {\rrbracket }_{0},0.4376\right) ,\left\langle 0.4253, 0.7780\right\rangle \right)$	$\left( \left( {\rrbracket }_{1},0.1233\right) ,\left\langle 0.5101, 0.7292\right\rangle \right)$

*Step 5* In view of Table 5 and Eq. (32), we derive the priority degrees for the criteria set. The outcomes are obtained as follows: 

*Step 6* Referring to Eq. (33), along with , we compute the evaluations for each alternative ${{\text{\Shilling}}}_i$ $(i = 1, 2, 3, 4, 5)$ using the Prioritized Sum Model $\bowtie _i^{(1)}$. The outcomes are presented in Table 7.

*Step 7* Following Eq. (34), , we determine the evaluations for each alternative ${{\text{\Shilling}}}_i$ using the prioritized product Model $\bowtie _i^{(2)}$ as tabulated in Table 7.

*Step 8* By applying Eq. (35), we combine the overall preference value $\bowtie _i$ for each alternative, as displayed in Table 7.

*Step 9* Considering the values of $\bowtie _i(i = 1, 2, 3, 4, 5)$, we arrange the alternatives in the following order: ${{\text{\Shilling}}}_5>{{\text{\Shilling}}}_3> {{\text{\Shilling}}}_1> {{\text{\Shilling}}}_4 > {{\text{\Shilling}}}_2$. As a result, the most significant patient is ${{\text{\Shilling}}}_5$.

**Table 6:** Measures for *q*-RF2LPMSM-based WASPAS.

	$\bowtie _i^{(1)}$	$\bowtie _i^{(2)}$	$\bowtie _i$
${{\text{\Shilling}}}_1$	0.1497	0.0056	0.0776
${{\text{\Shilling}}}_2$	0.1472	0.0047	0.0737
${{\text{\Shilling}}}_3$	0.1505	0.0060	0.0782
${{\text{\Shilling}}}_4$	0.1419	0.0045	0.0732
${{\text{\Shilling}}}_5$	0.1580	0.0071	0.0826

**Table 7:** Measures for *q*-RF2LPDMSM-based WASPAS.

	$\bowtie _i^{(1)}$	$\bowtie _i^{(2)}$	$\bowtie _i$
${{\text{\Shilling}}}_1$	0.1249	0.00008	0.0625
${{\text{\Shilling}}}_2$	0.1139	0.00002	0.0569
${{\text{\Shilling}}}_3$	0.1288	0.000006	0.0645
${{\text{\Shilling}}}_4$	0.1113	0.00001	0.0556
${{\text{\Shilling}}}_5$	0.1418	0.0001	0.0710

### Parameter analysis

This section presents an analysis of how sensitive the proposed model is to changes in the parameters $\theta$ and *q*, using the numerical example provided in “Background” section.

#### Sensitivity analysis with respect to $\theta$

**Figure 2:**
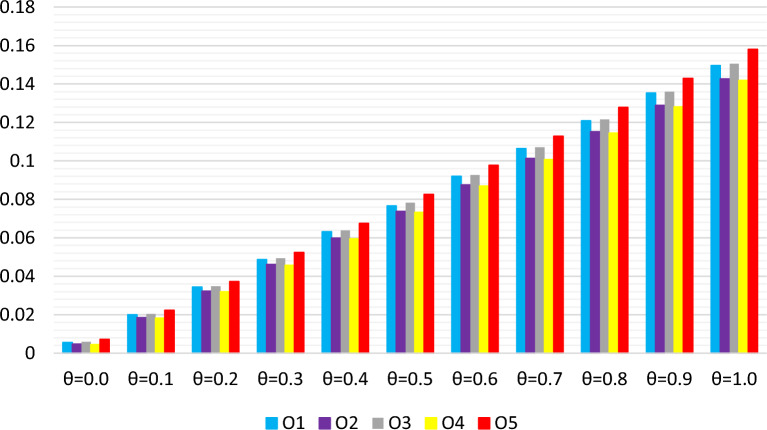
Ranking results via *q*-RF2LPMSM operator with different values of $\theta$.

The subsequent analysis focuses on understanding the impact of coefficient $\theta$ variations on our results. We employ the *q*-RF2LMSM-based WASPAS method to assess different scenarios in patient prioritization, considering a range of $\theta$ values from 0 to 1. The outcomes for each decision alternative ${{\text{\Shilling}}}_i$ $(i = 1, 2, 3, 4, 5$) at varying $\theta$ levels are detailed in Table 8. An examination of Table 8 and Fig. 2 reveals a trend where the values of $\bowtie _i$ ($i = 1, 2, 3, 4, 5$) rise with an increase in $\theta$ from 0 to 1. Notably, alternative ${{\text{\Shilling}}}_5$ consistently emerges as the top choice, indicating the robustness of the proposed method against changes in the strategy values set by experts.

**Figure 3:**
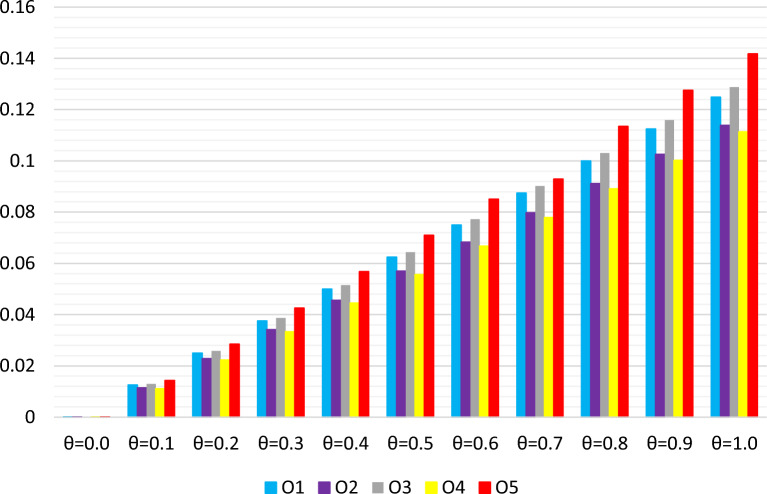
Ranking results via *q*-RF2LPDMSM operator with different values of $\theta$.

Next, we apply the *q*-RF2LDMSM-based WASPAS method to re-examine the patient prioritization decision-making process, varying $\theta$ within the 0 to 1 range. The outcomes for each option ${{\text{\Shilling}}}_i$ ($i = 1, 2, 3, 4, 5$) under these different $\theta$ values are shown in Table 9 and Fig. 3. Table 9 shows a similar pattern of increasing $\bowtie _i$ ($i = 1, 2, 3, 4, 5$) values as $\theta$ escalates from 0 to 1. Interestingly, alternative ${{\text{\Shilling}}}_5$ is again identified as the preferred option in all scenarios, affirming the stability of our method. The rankings of the other alternatives remain largely consistent, except the scenario where $\theta$ equals 0, further underscoring the resilience of the proposed approach amidst substantial modifications in expert strategy values.

**Table 8:** Ranking results of *q*-RF2LPMSM-based WASPAS with varying $\theta$ values.

$\theta$	${{\text{\Shilling}}}_1$	${{\text{\Shilling}}}_2$	${{\text{\Shilling}}}_3$	${{\text{\Shilling}}}_4$	${{\text{\Shilling}}}_5$	Ranking relation
$\theta =0.0$	0.00555	0.00469	0.00597	0.00450	0.00715	${{\text{\Shilling}}}_5>{{\text{\Shilling}}}_3>{{\text{\Shilling}}}_1>{{\text{\Shilling}}}_2>{{\text{\Shilling}}}_4$
$\theta =0.1$	0.01996	0.01849	0.02042	0.01824	0.02224	${{\text{\Shilling}}}_5>{{\text{\Shilling}}}_3>{{\text{\Shilling}}}_1>{{\text{\Shilling}}}_2>{{\text{\Shilling}}}_4$
$\theta =0.2$	0.03437	0.03229	0.03488	0.03198	0.03733	${{\text{\Shilling}}}_5>{{\text{\Shilling}}}_3>{{\text{\Shilling}}}_1>{{\text{\Shilling}}}_2>{{\text{\Shilling}}}_4$
$\theta =0.3$	0.04878	0.04610	0.04933	0.04571	0.05242	${{\text{\Shilling}}}_5>{{\text{\Shilling}}}_3>{{\text{\Shilling}}}_1>{{\text{\Shilling}}}_2>{{\text{\Shilling}}}_4$
$\theta =0.4$	0.06319	0.05990	0.06378	0.05942	0.06751	${{\text{\Shilling}}}_5>{{\text{\Shilling}}}_3>{{\text{\Shilling}}}_1>{{\text{\Shilling}}}_2>{{\text{\Shilling}}}_4$
$\theta =0.5$	0.07660	0.07370	0.07820	0.07320	0.08259	${{\text{\Shilling}}}_5>{{\text{\Shilling}}}_3>{{\text{\Shilling}}}_1>{{\text{\Shilling}}}_2>{{\text{\Shilling}}}_4$
$\theta =0.6$	0.09201	0.08750	0.09269	0.08693	0.09769	${{\text{\Shilling}}}_5>{{\text{\Shilling}}}_3>{{\text{\Shilling}}}_1>{{\text{\Shilling}}}_2>{{\text{\Shilling}}}_4$
$\theta =0.7$	0.10642	0.10131	0.10714	0.10067	0.11277	${{\text{\Shilling}}}_5>{{\text{\Shilling}}}_3>{{\text{\Shilling}}}_1>{{\text{\Shilling}}}_2>{{\text{\Shilling}}}_4$
$\theta =0.8$	0.12083	0.11511	0.12159	0.11440	0.12787	${{\text{\Shilling}}}_5>{{\text{\Shilling}}}_3>{{\text{\Shilling}}}_1>{{\text{\Shilling}}}_2>{{\text{\Shilling}}}_4$
$\theta =0.9$	0.13524	0.12891	0.13604	0.12814	0.14296	${{\text{\Shilling}}}_5>{{\text{\Shilling}}}_3>{{\text{\Shilling}}}_1>{{\text{\Shilling}}}_2>{{\text{\Shilling}}}_4$
$\theta =1.0$	0.14965	0.14271	0.15050	0.14188	0.15804	${{\text{\Shilling}}}_5>{{\text{\Shilling}}}_3>{{\text{\Shilling}}}_1>{{\text{\Shilling}}}_2>{{\text{\Shilling}}}_4$

**Table 9:** Ranking results of *q*-RF2LPDMSM-based WASPAS with varying $\theta$ values.

$\theta$	${{\text{\Shilling}}}_1$	${{\text{\Shilling}}}_2$	${{\text{\Shilling}}}_3$	${{\text{\Shilling}}}_4$	${{\text{\Shilling}}}_5$	Ranking relation
$\theta =0.0$	0.00008	0.00002	0.00006	0.00001	0.0001	${{\text{\Shilling}}}_5>{{\text{\Shilling}}}_1>{{\text{\Shilling}}}_3>{{\text{\Shilling}}}_2>{{\text{\Shilling}}}_4$
$\theta =0.1$	0.01256	0.01141	0.01293	0.01114	0.01427	${{\text{\Shilling}}}_5>{{\text{\Shilling}}}_3>{{\text{\Shilling}}}_1>{{\text{\Shilling}}}_2>{{\text{\Shilling}}}_4$
$\theta =0.2$	0.02504	0.02279	0.02581	0.02227	0.02844	${{\text{\Shilling}}}_5>{{\text{\Shilling}}}_3>{{\text{\Shilling}}}_1>{{\text{\Shilling}}}_2>{{\text{\Shilling}}}_4$
$\theta =0.3$	0.03753	0.03418	0.03868	0.03334	0.04261	${{\text{\Shilling}}}_5>{{\text{\Shilling}}}_3>{{\text{\Shilling}}}_1>{{\text{\Shilling}}}_2>{{\text{\Shilling}}}_4$
$\theta =0.4$	0.05001	0.04557	0.05155	0.04452	0.05678	${{\text{\Shilling}}}_5>{{\text{\Shilling}}}_3>{{\text{\Shilling}}}_1>{{\text{\Shilling}}}_2>{{\text{\Shilling}}}_4$
$\theta =0.5$	0.06249	0.05696	0.06443	0.05565	0.07095	${{\text{\Shilling}}}_5>{{\text{\Shilling}}}_3>{{\text{\Shilling}}}_1>{{\text{\Shilling}}}_2>{{\text{\Shilling}}}_4$
$\theta =0.6$	0.07497	0.06835	0.07730	0.06678	0.08512	${{\text{\Shilling}}}_5>{{\text{\Shilling}}}_3>{{\text{\Shilling}}}_1>{{\text{\Shilling}}}_2>{{\text{\Shilling}}}_4$
$\theta =0.7$	0.08745	0.07973	0.09018	0.07791	0.09290	${{\text{\Shilling}}}_5>{{\text{\Shilling}}}_3>{{\text{\Shilling}}}_1>{{\text{\Shilling}}}_2>{{\text{\Shilling}}}_4$
$\theta =0.8$	0.09999	0.09112	0.10305	0.08904	0.11346	${{\text{\Shilling}}}_5>{{\text{\Shilling}}}_3>{{\text{\Shilling}}}_1>{{\text{\Shilling}}}_2>{{\text{\Shilling}}}_4$
$\theta =0.9$	0.11242	0.10251	0.11593	0.10017	0.12760	${{\text{\Shilling}}}_5>{{\text{\Shilling}}}_3>{{\text{\Shilling}}}_1>{{\text{\Shilling}}}_2>{{\text{\Shilling}}}_4$
$\theta =1.0$	0.12490	0.11390	0.12880	0.11130	0.14180	${{\text{\Shilling}}}_5>{{\text{\Shilling}}}_3>{{\text{\Shilling}}}_1>{{\text{\Shilling}}}_2>{{\text{\Shilling}}}_4$

#### Sensitivity analysis with respect to *q*

**Figure 4:**
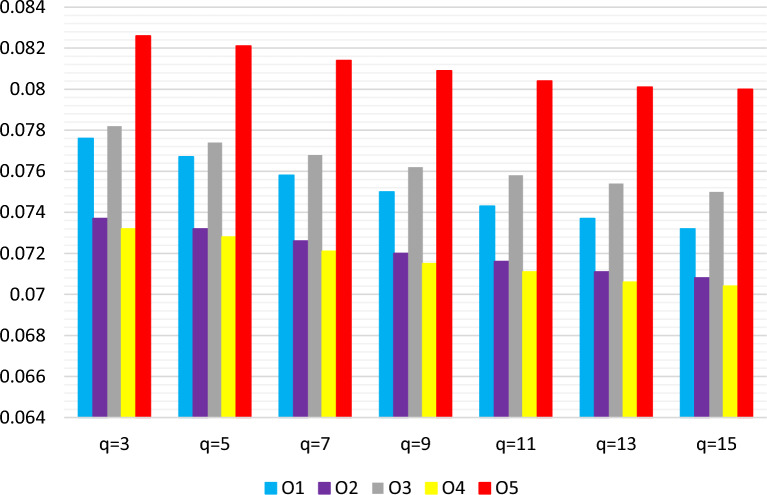
Ranking results via *q*-RF2LPMSM operator with different values of *q*.

This section examines the impact of the parameter *q* on the results. Initially, we alter the *q*values within the *q*-RF2LPMSM-based WASPAS framework, keeping the other parameter, $\theta$, constant at 0.5. The results, summarized in Table 10 and Fig. 4, show a distinct trend: as *q* increases, the scores for all alternatives decrease. However, the overall ranking of alternatives remains unaffected by changes in *q*.

**Figure 5:**
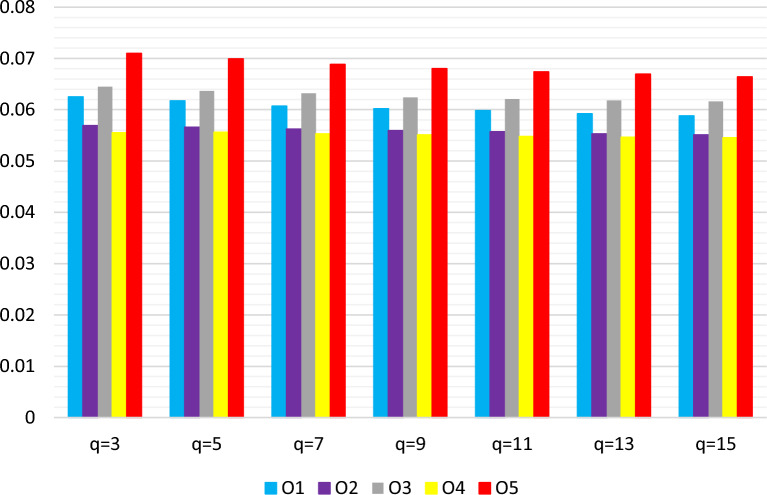
Ranking results via *q*-RF2DLMSM operator with different values of *q*.

Similarly, a comparable pattern emerges when adjusting *q* within the *q*-RF2LPDMSM-based WASPAS method: scores fluctuate with changes in *q*. Yet, the ranking order of alternatives stays consistent (see Table 11 and Fig. 5). This consistency across different *q* values highlights the robustness and stability of the proposed approach about its parameters.

**Table 10:** Ranking results of *q*-RF2LPMSM-based WASPAS with varying *q* values.

*q*	${{\text{\Shilling}}}_1$	${{\text{\Shilling}}}_2$	${{\text{\Shilling}}}_3$	${{\text{\Shilling}}}_4$	${{\text{\Shilling}}}_5$	Ranking relation
3	0.0776	0.0737	0.0782	0.0732	0.0826	${{\text{\Shilling}}}_5>{{\text{\Shilling}}}_3>{{\text{\Shilling}}}_1>{{\text{\Shilling}}}_2>{{\text{\Shilling}}}_4$
5	0.0767	0.0732	0.0774	0.0728	0.0821	${{\text{\Shilling}}}_5>{{\text{\Shilling}}}_3>{{\text{\Shilling}}}_1>{{\text{\Shilling}}}_2>{{\text{\Shilling}}}_4$
7	0.0758	0.0726	0.0768	0.0721	0.0814	${{\text{\Shilling}}}_5>{{\text{\Shilling}}}_3>{{\text{\Shilling}}}_1>{{\text{\Shilling}}}_2>{{\text{\Shilling}}}_4$
9	0.0750	0.0720	0.0762	0.0715	0.0809	${{\text{\Shilling}}}_5>{{\text{\Shilling}}}_3>{{\text{\Shilling}}}_1>{{\text{\Shilling}}}_2>{{\text{\Shilling}}}_4$
11	0.0743	0.0716	0.0758	0.0711	0.0804	${{\text{\Shilling}}}_5>{{\text{\Shilling}}}_3>{{\text{\Shilling}}}_1>{{\text{\Shilling}}}_2>{{\text{\Shilling}}}_4$
13	0.0737	0.0711	0.0754	0.0706	0.0801	${{\text{\Shilling}}}_5>{{\text{\Shilling}}}_3>{{\text{\Shilling}}}_1>{{\text{\Shilling}}}_2>{{\text{\Shilling}}}_4$
15	0.0732	0.0708	0.0750	0.0704	0.0800	${{\text{\Shilling}}}_5>{{\text{\Shilling}}}_3>{{\text{\Shilling}}}_1>{{\text{\Shilling}}}_2>{{\text{\Shilling}}}_4$

**Table 11:** Ranking results of *q*-RF2LPDMSM-based WASPAS with varying *q* values.

*q*	${{\text{\Shilling}}}_1$	${{\text{\Shilling}}}_2$	${{\text{\Shilling}}}_3$	${{\text{\Shilling}}}_4$	${{\text{\Shilling}}}_5$	Ranking relation
3	0.0625	0.0569	0.0645	0.0555	0.0710	${{\text{\Shilling}}}_5>{{\text{\Shilling}}}_3>{{\text{\Shilling}}}_1>{{\text{\Shilling}}}_2>{{\text{\Shilling}}}_4$
5	0.0617	0.0566	0.0637	0.0556	0.0699	${{\text{\Shilling}}}_5>{{\text{\Shilling}}}_3>{{\text{\Shilling}}}_1>{{\text{\Shilling}}}_2>{{\text{\Shilling}}}_4$
7	0.0607	0.0562	0.0632	0.0553	0.0688	${{\text{\Shilling}}}_5>{{\text{\Shilling}}}_3>{{\text{\Shilling}}}_1>{{\text{\Shilling}}}_2>{{\text{\Shilling}}}_4$
9	0.0602	0.0559	0.0624	0.0551	0.0680	${{\text{\Shilling}}}_5>{{\text{\Shilling}}}_3>{{\text{\Shilling}}}_1>{{\text{\Shilling}}}_2>{{\text{\Shilling}}}_4$
11	0.0598	0.0557	0.0621	0.0548	0.0674	${{\text{\Shilling}}}_5>{{\text{\Shilling}}}_3>{{\text{\Shilling}}}_1>{{\text{\Shilling}}}_2>{{\text{\Shilling}}}_4$
13	0.0592	0.0553	0.0618	0.0546	0.0669	${{\text{\Shilling}}}_5>{{\text{\Shilling}}}_3>{{\text{\Shilling}}}_1>{{\text{\Shilling}}}_2>{{\text{\Shilling}}}_4$
15	0.0588	0.0551	0.0616	0.0545	0.0664	${{\text{\Shilling}}}_5>{{\text{\Shilling}}}_3>{{\text{\Shilling}}}_1>{{\text{\Shilling}}}_2>{{\text{\Shilling}}}_4$

## Validation of results

This section evaluates the proposed model’s comparability with existing methodologies, highlighting its efficiency and superiority. Given the limited number of studies that utilize multi-criteria modeling methods like WASPAS in conjunction with prioritized operators, our options for comparison with MCGDM approaches are somewhat restricted. Consequently, we undertake a step-by-step comparison of the proposed approach against each existing method.

### *q*-RF2L PROMETHEE II method

In this section, we contrast our developed approach with the *q*-RF2L preference ranking organization method for enrichment evaluation (PROMETHEE) II technique, as introduced by Li et al.^48^. This comparison aims to assess the logical validity of our method, beginning with the aggregated decision matrix presented in Table 4.

Based on Eq. (36), the deviations between the two assessment values are calculated in Table 11.36where  is the Hamming distance between  and ^48^.37

**Table 12:** Deviations of pairwise evaluations.

	€$_1$	€$_2$	€$_3$	€$_4$
	1.5124	0.0000	1.3771	0.0000
	0.0000	1.4568	0.3521	0.0420
	1.7063	0.0000	0.0000	1.4499
	0.0000	0.3028	2.5221	0.0528
	0.0000	0.3069	0.0000	0.8057
	0.0000	1.7637	0.0000	0.8478
	0.1939	0.2438	0.0000	2.2557
	0.0000	0.6097	1.1449	0.8585
	0.4821	0.0000	0.0000	0.0000
	1.9945	0.0000	1.0251	0.0000
	2.1884	0.0000	0.0000	1.4079
	0.0000	0.0000	2.1699	0.0000
	0.0000	0.0631	0.2053	0.0000
	0.0000	0.0000	1.5826	0.0000
	0.0000	1.5199	0.5574	0.0000
	0.0000	0.3659	2.7274	0.0000
	1.6088	0.0000	0.0000	0.0000
	3.1212	0.0000	0.0000	0.0000
	1.1267	1.1540	0.0000	0.0108
	3.3151	0.0000	0.0000	1.3971

where . Here, $f\left( \cdot \right)$ is the preference function.

Following Eq. (37), the global preference of each patient is conducted shown follows:

38 $$\begin{aligned}{} & {} \psi ^{+}\left( {{\text{\Shilling}}}_i\right) =\frac{1}{m-1}\sum {\coprod }({{\text{\Shilling}}}_i,{{\text{\Shilling}}}_r){{,}} \end{aligned}$$

39 $$\begin{aligned}{} & {} \psi ^{-}\left( {{\text{\Shilling}}}_i\right) =\frac{1}{m-1}\sum {\coprod }({{\text{\Shilling}}}_r,{{\text{\Shilling}}}_i){{.}} \end{aligned}$$

According to Eqs. (38) and (39), the positive flow $\Psi ^{+}({{\text{\Shilling}}}_i)$ and negative flow $\Psi ^{-}({{\text{\Shilling}}}_i)$ of each option are calculated, respectively, as given below:

$$\Psi ^{+}({{\text{\Shilling}}}_1)=0.3429,$$ $$\Psi ^{+}({{\text{\Shilling}}}_2)=0.2217,$$ $$\Psi ^{+}({{\text{\Shilling}}}_3)=0.3161,$$ $$\Psi ^{+}({{\text{\Shilling}}}_4)=0.2042,$$ $$\Psi ^{+}({{\text{\Shilling}}}_5)=0.3456.$$

$$\Psi ^{-}({{\text{\Shilling}}}_1)=0.1641,$$ $$\Psi ^{-}({{\text{\Shilling}}}_2)=0.3645,$$ $$\Psi ^{-}({{\text{\Shilling}}}_3)=0.2746,$$ $$\Psi ^{+}({{\text{\Shilling}}}_4)=0.3753,$$ $$\Psi ^{-}({{\text{\Shilling}}}_5)=0.2944.$$

Using the values of $\Psi ^{+}({{\text{\Shilling}}}_i)$ and $\Psi ^{-}({{\text{\Shilling}}}_i)$, the net flows $\Psi ({{\text{\Shilling}}}_i)= \psi ^{+}\left( {{\text{\Shilling}}}_i\right) - \psi ^{-}\left( {{\text{\Shilling}}}_i\right)$ for each alternative are determined as follows:

$$\Psi ({{\text{\Shilling}}}_1)=0.1789,$$ $$\Psi ({{\text{\Shilling}}}_2)=-0.1428,$$ $$\Psi ({{\text{\Shilling}}}_3)=0.0415,$$ $$\Psi ({{\text{\Shilling}}}_4)=-0.1711,$$ $$\Psi ({{\text{\Shilling}}}_5)=0.0512.$$

Thus, the ranking with *q*-RF2L PROMETHEE II method^48^ is ${{\text{\Shilling}}}_1>{{\text{\Shilling}}}_5>{{\text{\Shilling}}}_3>{{\text{\Shilling}}}_2>{{\text{\Shilling}}}_4.$

### *q*-RFOL Muirhead mean aggregation operators-based method

This section focuses on obtaining results using the *q*-RF2L Muirhead mean (*q*-RF2LMM) AOs-based method, as detailed in^34^. The process is outlined through the following steps:

To aggregate *q*-RF2L assessment values  for each alternative ${{\text{\Shilling}}}_i$ across all criteria  into a single overall assessment value $\partial _i$, as shown in Table 4, we utilize the *q*-RF2LMM operator. This operator is detailed in Eq. (40), and is applied with the parameter $P=(1,1,2,1)$ to aggregate the values for each alternative ${{\text{\Shilling}}}_i(i = 1, 2, 3, 4,5)$ (Table 12).40where $P=(P_1,P_2,...,P_{{\scalebox{0.7}{\text{\saturn}}}} )$ is a vector of parameters and  is a permutation of $(1, 2, \ldots , {{\scalebox{0.7}{\text{\saturn}}}})$ and $S_{{\scalebox{0.7}{\text{\saturn}}}}$ is a set of all permutations of $(1, 2, \ldots , {{\scalebox{0.7}{\text{\saturn}}}})$.

After the implication of Eq. (41) the overall assessment values of alternatives ${{\text{\Shilling}}}_i (i = 1, 2, 3, 4,5)$ are obtained as:

$$\begin{aligned}{} & {} \partial _1=\left( \left( {\rrbracket }_2,-0.4676\right) ,\left\langle 0.8509,0.2654 \right\rangle \right) , \partial _2=\left( \left( {\rrbracket }_1,0.3420 \right) ,\left\langle 0.9007,0.2574 \right\rangle \right) , \\{} & {} \partial _3=\left( \left( {\rrbracket }_1,0.3719 \right) ,\left\langle 0.8925,0.1619 \right\rangle \right) , \partial _4=\left( \left( {\rrbracket }_1,0.3198 \right) ,\left\langle 0.8835,0.1949 \right\rangle \right) , \\{} & {} \partial _5=\left( \left( {\rrbracket }_1,0.3749 \right) ,\left\langle 0.8944,0.2230 \right\rangle \right) . \end{aligned}$$

According to Eq. (6), the score values of overall assessment value $\partial _i (i=1,2,3,4,5)$ are determined as given below:

$$\begin{aligned} {\text{\t{S}}(\partial_1)}=0.1748, {\text{\t{S}}(\partial_2)}=0.1643, {\text{\t{S}}(\partial_3)}=0.1672, {\text{\t{S}}(\partial_4)}=0.1586, {\text{\t{S}}(\partial_5)}=0.1674{{.}} \end{aligned}$$

Based on the above score values, the ranking of all patients is obtained: ${{\text{\Shilling}}}_1>{{\text{\Shilling}}}_5>{{\text{\Shilling}}}_3>{{\text{\Shilling}}}_2>{{\text{\Shilling}}}_4.$

Based on the outcome provided above, it is evident that there exists a minor distinction in the ranking of the two approaches, i.e., the alternative ${{\text{\Shilling}}}_1$ only changes the position while the remaining alternatives have the same rank.

### *q*-Rung orthopair fuzzy Macularin symmetric mean operators-based method

The effectiveness of the proposed method is further substantiated through a comparative analysis with the *q*-rung orthopair Maclaurin symmetric mean (*q*-ROFMSM) operators-based method created by Wei et al.^49^. In order to align the Wei et al. method with the specific issue at hand, we have excluded the linguistic terms and their corresponding symbolic translations, as detailed in the data presented in Table 4. This modification enables us to derive the subsequent calculated results, which are comprehensively displayed in Table 13.41
where $\mathfrak {a}_{i}= \left\langle {{\mathfrak {m}}}_i, {{\mathfrak {n}}}_i\right\rangle \left( i=1,2, \ldots ,{{\scalebox{0.7}{\text{\saturn}}}} \right)$ represent family of *q*-ROFNs, , and .

**Table 13:** *q*-rung orthopair fuzzy group decision matrix.

	$c_1$	$c_2$	$c_3$	$c_4$
${{\text{\Shilling}}}_1$	$\left\langle 0.8796,0.2415 \right\rangle$	$\left\langle 0.8171,0.2991 \right\rangle$	$\left\langle 0.8326,0.2924 \right\rangle$	$\left\langle 0.8735,0.2093 \right\rangle$
${{\text{\Shilling}}}_2$	$\left\langle 0.8729,0.2348 \right\rangle$	$\left\langle 0.8749,0.2295 \right\rangle$	$\left\langle 0.9162, 0.1942\right\rangle$	$\left\langle 0.9185,0.3314 \right\rangle$
${{\text{\Shilling}}}_3$	$\left\langle 0.9011,0.1420 \right\rangle$	$\left\langle 0.9455,0.2347 \right\rangle$	$\left\langle 0.8303,0.0876 \right\rangle$	$\left\langle 0.8946,0.0951 \right\rangle$
${{\text{\Shilling}}}_4$	$\left\langle 0.8822,0.2127 \right\rangle$	$\left\langle 0.8964, 0.1647\right\rangle$	$\left\langle 0.8709,0.2378 \right\rangle$	$\left\langle 0.8838,0.1307 \right\rangle$
${{\text{\Shilling}}}_5$	$\left\langle 0.8453,0.2623 \right\rangle$	$\left\langle 0.9129, 0.1944\right\rangle$	$\left\langle 0.9153,0.1633 \right\rangle$	$\left\langle 0.9048,0.2460 \right\rangle$

By employing the *q*-ROFMSM operator (see Eq. (41)), the overall assessment values for the alternatives ${{\text{\Shilling}}}_i (i = 1, 2, 3, 4,5)$ are calculated as follows:

$$\begin{aligned} \mathfrak {a}_1=\left\langle 0.8519,0.2632 \right\rangle , \mathfrak {a}_2=\left\langle 0.9014,0.2513 \right\rangle , \mathfrak {a}_3=\left\langle 0.8957,0.1462 \right\rangle , \mathfrak {a}_4=\left\langle 0.8836,0.1909 \right\rangle , \mathfrak {a}_5=\left\langle 0.8959,0.2199 \right\rangle . \end{aligned}$$

Based on the score function of q-ROFNs^19^, the score values of overall assessment value $\mathfrak {a}_i (i=1,2,3,4,5)$ are determined as outlined below:

$$\begin{aligned} S(\mathfrak {a}_1)=0.8000, S(\mathfrak {a}_2)=0.8502, S(\mathfrak {a}_3)=0.8578, S(\mathfrak {a}_4)=0.8415, S(\partial _5)=0.8543. \end{aligned}$$

Based on the above score values, the ranking of all patients is obtained: ${{\text{\Shilling}}}_3>{{\text{\Shilling}}}_5>{{\text{\Shilling}}}_2>{{\text{\Shilling}}}_4>{{\text{\Shilling}}}_1.$

From Table 12, it can be seen that all the ranking results of the two approaches are different except ${{\text{\Shilling}}}_4$. The difference is due to the absence of linguistic terms in the approach of Wei et al.^49^. It is basically developed on *q*-ROFSs, which only process the uncertain data in quantitative form. This technique is unsuitable for modeling decision information when DMs prefer utilizing linguistic values.

**Table 14:** Decision results obtained by using different methods.

Method	Ranking results
*q*-RFL PROMETHEE II method^48^	${{\text{\Shilling}}}_1>{{\text{\Shilling}}}_5>{{\text{\Shilling}}}_3>{{\text{\Shilling}}}_2>{{\text{\Shilling}}}_4$
*q*-RF2LMM AOs-based method^34^	${{\text{\Shilling}}}_1>{{\text{\Shilling}}}_5>{{\text{\Shilling}}}_3>{{\text{\Shilling}}}_2>{{\text{\Shilling}}}_4$
*q*-ROFMSM operators-based method^49^	${{\text{\Shilling}}}_3>{{\text{\Shilling}}}_5>{{\text{\Shilling}}}_2>{{\text{\Shilling}}}_4>{{\text{\Shilling}}}_1$
Proposed method	${{\text{\Shilling}}}_5>{{\text{\Shilling}}}_3>{{\text{\Shilling}}}_1>{{\text{\Shilling}}}_4>{{\text{\Shilling}}}_2$

### Discussion

(i): Analysis of Table 14 reveals that alternative ${{\text{\Shilling}}}_5$, initially deemed optimal, ranks second when evaluated through existing methodologies. Specifically, in the method proposed by Lie et al.^48^, the q-RF2LWA operator is utilized for the aggregation phase in decision-making. A notable limitation of this operator is its failure to account for the interrelationships among multiple input arguments. Additionally, the Lie et al. method lacks mechanisms to prioritize criteria and experts. Although it includes weight determination strategies, these are not sufficient for practical problems where prioritization of both criteria and DMs is crucial. In such contexts, our proposed approach demonstrates superior adaptability and effectiveness.
(ii): When compared with the Ju et al. method^34^, it’s evident that the overall rankings diverge from those obtained using our proposed approach. This discrepancy primarily arises because the existing approach focuses solely on the interrelationship among input arguments, as evident from Eq. (40). It also overlooks the crucial aspect of modeling the prioritization relationships among experts and the set criteria. In contrast, our proposed methods, which leverage the q-RF2LPMSM and q-RF2LPDMSM, adeptly handle the interrelationships among multiple input arguments and duly consider the prioritization among both DMs and criteria. Additionally, our method integrates the q-RF2LPMSM and q-RF2LPDMSM operators within the WASPAS method framework. These distinctive features render our proposed methods notably more practical and adaptable.
(iii): From Table 14, we can see that most of the alternative ranking positions obtained via the Wei et al.^49^ approach is inconsistent with that obtained through our proposed method. This discrepancy primarily stems from the fact that the operator in Eq. (41) evaluates alternatives using q-rung orthopair fuzzy data but disregards linguistic terms and their symbolic translations. In contrast, the q-RF2LPMSM and q-RF2LPDMSM operators introduced in our study focus on q-RF2L data and effectively incorporate these linguistic aspects. When the linguistic terms and their symbolic translations are omitted, these operators essentially become the q-rung orthopair fuzzy prioritized MSM and DMSM operators, respectively. The inclusion of linguistic terms and symbolic translations, along with the prioritization feature, enables our operators to mirror the subjective perspectives of DMs more accurately. The omission of these aspects in Wei et al.’s approach results in a loss of crucial information, potentially leading to erroneous outcomes. Furthermore, the approach of Wei et al. is limited to MCDM problems and does not adequately address DM scenarios involving multiple experts.

From the preceding discussion, the key advantages of our initiated approach are summarized as follows:

1. The foremost benefit of our proposed approach lies in the design of the formulated operators, which adeptly incorporate interdependencies among arguments. Additionally, these operators facilitate prioritization among criteria and DMs within the DM process.
2. The proposed method demonstrates robust stability, even with significant adjustments made to the involved parameters, $\theta$ and *q*. This stability is evidenced by the consistent ranking order, as detailed in “5.3” section.
3. The AOs developed in our study (Eqs. (13) and (21)) are advanced generalizations of numerous existing AOs. For details and insights into their extended capabilities, please refer to “3.1” and “3.2” sections.
4. Unlike the existent methods^34,49^, our developed approach is not solely reliant on AOs. It innovatively integrates the formulated operators, q-RF2LPMSM and q-RF2LPDMSM, with the WASPAS method within a q-RF2L environment. This integration significantly enhances the adaptability and applicability of our methodology, setting it apart from existing methods.

In addition to the aforementioned advantages, it is noteworthy that the proposed prioritized AOs exhibit certain limitations. Specifically, these AOs lack the property of idempotency, wherein the weighted average value of equivalent fuzzy numbers fluctuates with their prioritized weights. Moreover, the designed AOs do not conform to the MSM/DMSM when their prioritized weights are equal, thus indicating a deficiency in reducibility property.

## Concluding remarks

The pressing need for efficient allocation of limited medical resources has led many healthcare facilities to prioritize patient hospitalization. In response, this paper introduced a novel MCGDM technique to address patient prioritization effectively. This technique, which acknowledges the importance of prioritization relationships, integrates the MSM and the DMSM operators with the PA operator, leading to the development of the q-RF2LPMSM and q-RF2LPDMSM operators. We have thoroughly verified the key characteristics of these newly proposed prioritized aggregation operators and examined various special cases. Building on these operators, we have enhanced the WASPAS method to create an innovative ranking mechanism tailored for q-RF2L MCGDM challenges. The q-RF2LPMSM and q-RF2LPDMSM operators are not only adept at managing the interdependence among multiple input parameters but also effectively consider the prioritization among experts and criteria, reflecting the experts’ attitudinal perspectives. The created method is highly flexible, which makes it suitable for resolving similar prioritization challenges that utilize q-RF2L information for evaluating alternatives. However, this study has a limitation in that it does not consider the weights of DMs and the absence of a consensus-building model among them. Future research should aim to improve information aggregation by determining the weights of DMs and refining the formulations of prioritized operators to ensure compliance with the properties of idempotency and reducibility.

## Data Availability

All data generated or analysed during this study are included in this published article.
